# ApoC-III ASO promotes tissue LPL activity in the absence of apoE-mediated TRL clearance[S]

**DOI:** 10.1194/jlr.M093740

**Published:** 2019-05-14

**Authors:** Bastian Ramms, Sohan Patel, Chelsea Nora, Ariane R. Pessentheiner, Max W. Chang, Courtney R. Green, Gregory J. Golden, Patrick Secrest, Ronald M. Krauss, Christian M. Metallo, Christopher Benner, Veronica J. Alexander, Joseph L. Witztum, Sotirios Tsimikas, Jeffrey D. Esko, Philip L. S. M. Gordts

**Affiliations:** Departments of Cellular and Molecular Medicine,* University of California, San Diego, La Jolla, CA; Medicine,† University of California, San Diego, La Jolla, CA; Bioengineering,†† University of California, San Diego, La Jolla, CA; Glycobiology Research and Training Center,§ University of California, San Diego, La Jolla, CA; Department of Chemistry,** Biochemistry I, Bielefeld University, Bielefeld, Germany; Children’s Hospital Oakland Research Institute§§ Oakland, CA; Ionis Pharmaceuticals Inc.,*** Carlsbad, CA

**Keywords:** lipid metabolism, apolipoprotein C-III, apolipoprotein E, triglyceride-rich lipoprotein clearance, fatty acids, lipase, lipoprotein lipase

## Abstract

Hypertriglyceridemia results from accumulation of triglyceride (TG)-rich lipoproteins (TRLs) in the circulation and is associated with increased CVD risk. ApoC-III is an apolipoprotein on TRLs and a prominent negative regulator of TG catabolism. We recently established that in vivo apoC-III predominantly inhibits LDL receptor-mediated and LDL receptor-related protein 1-mediated hepatic TRL clearance and that apoC-III-enriched TRLs are preferentially cleared by syndecan-1 (SDC1). In this study, we determined the impact of apoE, a common ligand for all three receptors, on apoC-III metabolism using apoC-III antisense oligonucleotide (ASO) treatment in mice lacking apoE and functional SDC1 (*Apoe*^−/−^*Ndst1*^f/f^*Alb-Cre*^+^). ApoC-III ASO treatment significantly reduced plasma TG levels in *Apoe*^−/−^*Ndst1*^f/f^*Alb-Cre*^+^ mice without reducing hepatic VLDL production or improving hepatic TRL clearance. Further analysis revealed that apoC-III ASO treatment lowered plasma TGs in *Apoe*^−/−^*Ndst1*^f/f^*Alb-Cre*^+^ mice, which was associated with increased LPL activity in white adipose tissue in the fed state. Finally, clinical data confirmed that ASO-mediated lowering of APOC-III via volanesorsen can reduce plasma TG levels independent of the APOE isoform genotype. Our data indicate that apoE determines the metabolic impact of apoC-III as we establish that apoE is essential to mediate inhibition of TRL clearance by apoC-III and that, in the absence of functional apoE, apoC-III inhibits tissue LPL activity.

Elevated plasma triglyceride (TG) levels are an independent risk factor for CVD and all-cause mortality (1). The concentration of plasma TG levels reflects a balance between de novo synthesis in the liver (VLDLs), intestinal absorption of dietary lipids (chylomicrons), lipolysis in the peripheral circulation, and hepatic clearance. TG-rich lipoproteins (TRLs) carry TGs in the blood and are rapidly hydrolyzed by LPL, thereby releasing free FAs for energy production or storage in the surrounding tissues (2–4). The remnant TRLs are subsequently rapidly cleared in the liver by the interaction of apolipoproteins on TRLs with the three main hepatic receptors, heparan sulfate proteoglycan syndecan-1 (SDC1), LDL receptor (LDLR), and LDLR-related protein 1 (LRP1) (5).

TRLs carry several apolipoproteins, including apoB, apoE, apoAV, and apoC-III. ApoB and apoE serve as ligands for LDLR and LRP1, promoting hepatic TLR clearance (6–10). In contrast, hepatic SDC1 recognizes apoE and apoAV, dependent on the interaction of these apolipoproteins with the heparan sulfate side chains on SDC1 (9). Human apoE is a 299 amino acid polymorphic glycoprotein synthesized and secreted primarily by liver, brain, skin, and macrophages (11). In humans, three isoforms, apoE4 (A112/A158), apoE3 (C112/A158), and apoE2 (C112/C158), differ by single amino acid substitutions at two key nonsynonymous sites. ApoE3 and apoE4 can bind to LDLR and LRP1, whereas apoE2 does not. ApoC-III is an 8.8 kDa glycoprotein present on all lipoprotein classes and one of the key modulators of TG metabolism (12). Transgenic expression of *Apoc3* results in hypertriglyceridemia in mice (13), whereas gene targeted deletion of *Apoc3* decreases TG levels (14). The importance of apoC-III in humans was established by the findings that inactivating mutations were shown to correlate with lower plasma TGs (15) and to protect against CVD (16–18). Depressing APOC-III expression using an antisense oligonucleotide (ASO), volanesorsen, also reduces plasma TGs in mildly hypertriglyceridemic patients (5, 19–21). In a recent clinical study, treatment of massively hypertriglyceridemic LPL-deficient patients (<5% LPL activity) with volanesorsen resulted in profound lowering of plasma TG levels as well, consistent with the idea that apoC-III also modulates plasma TG levels in a non-LPL-dependent manner, likely via LDLR/LRP1-mediated clearance (5, 19).

It remains unclear exactly how apoC-III blocks LRP1- and LDLR-mediated TRL clearance. Direct inhibition of apoC-III on apoE-mediated TRL binding to LDLR and LRP1 as well as the competition between apoE and apoC-III for space on TRLs have been proposed as mechanisms (13, 14, 22–24). However, intercrossing *Apoc3*^−/−^ mice with *Apoe*^−/−^ mice resulted in a marked reduction in VLDL TG, indicating that apoC-III deficiency exerts its lipid-lowering effect independently of apoE (25). At the time these studies were underway, the participation of SDC1-mediated clearance was not fully appreciated.

Thus, we set out to analyze the impact of suppressing apoC-III expression on TRL clearance and lipid levels in the absence of apoE and SDC1-mediated TRL clearance. We probed this question in mice lacking functional SDC1 by liver-specific targeted inactivation of *Ndst1*, an enzyme involved in formation of the heparan sulfate chains compounded by apoE-inactivation (*Apoe*^−/−^*Ndst1*^f/f^*Alb-Cre*^+^). Hence, both apoE-mediated TRL clearance through LDLR/LRP1- and SDC1-mediated TRL clearance were abolished. Based on our previous studies showing that apoC-III targeting lowered TG levels independent of LPL expression, we anticipated to find no or only a minor effect of targeting apoC-III on plasma TG levels due to the lack of TRL clearance (5, 19). However, we observed that administration of apoC-III ASO to *Apoe*^−/−^*Ndst1*^f/f^*Alb-Cre*^+^ mice dramatically reduced TG levels, independently of TRL clearance or hepatic VLDL production. Plasma and heparin-releasable LPL levels were unaffected in this model as well. Further analysis showed that apoC-III lowering in the absence of apoE expression elevated LPL activity and TG hydrolysis in white adipose tissue (WAT). We also show that the administration of APOC-III ASO to patients significantly reduced plasma TGs independently of the APOE isoform, even in APOE2 homozygous patients, which is in line with the observed apoC-III ASO-mediated TG-lowering in mice.

## METHODS

### Mice

*Apoe*^−/−^,*Alb-Cre*^+^ and *Ldlr*^−/−^ mice were purchased from the Jackson Laboratory. *Ndst1*^f/f^*Alb-Cre*^+^, *Apoe*^−/−^*Ndst1*^f/f^*Alb-Cre*^+^, and *Ldlr*^−/−^*Ndst1*^f/f^*Alb-Cre*^+^ mice were generated and genotyped as described (26, 27). All animals were fully backcrossed onto the C57Bl/6 background. All animals were housed and bred in vivaria approved by the Association for Assessment and Accreditation of Laboratory Animal Care located in the School of Medicine, University of California, San Diego, following standards and procedures approved by the University of California, San Diego Institutional Animal Care and Use Committee. Mice were weaned at 4 weeks, maintained on a 12 h light cycle, and fed ad libitum with water and standard rodent chow (PicoLab® Rodent Diet 20 5053) or a Western diet (WD) (TD.88137; Envigo Teklad). Mice received ION 440726 (murine apoC-III ASO) or ION 141923 (murine control ASO) at 50 mg/kg/week (supplemental Table S1) via intraperitoneal injections.

### Lipid analysis

Lipid levels were analyzed in plasma and liver samples. Blood was drawn via the tail vein from mice fasted for 5 h. Total plasma cholesterol and TG levels were determined using commercially available kits (Sekisui Diagnostics). Cholesterol in VLDL fractions was measured with the Amplex Red cholesterol assay kit (Thermo Fisher Scientific). Plasma NEFA and 3-hydroxybutyrate levels were determined using enzymatic kits (Wako Chemicals). Liver samples were homogenized using a hypotonic extraction buffer [250 mM sucrose, 5 mM Tris, 1 mM EDTA, and proteinase inhibitor (cOmplete; Sigma)] and total cholesterol and TG were determined in the supernatant after centrifugation as described above.

### RNA analysis

Total RNA was isolated with Trizol from homogenized tissue and cells and purified using RNeasy columns and RNase-free DNase digestion according to the manufacturer’s instructions (Qiagen). The quality and quantity of the total RNA was monitored and measured via NanoDrop (NanoDrop Technologies, Inc., Wilmington, DE) following the manufacturer’s instructions. For quantitative PCR analysis, 1 μl of cDNA was used for real-time PCR with gene-specific primers (supplemental Table S2) and *Tbp* as a housekeeping gene on a Bio-Rad CFX96 real-time PCR system (Bio-Rad).

### Fast-performance LC

Plasma was pooled from several mice (100 μl per mouse, n = 3–5 mice per genotype) and separated by gel filtration fast-performance (FP)LC using a GE Superose 6 10/300 GL column in 0.15 M sodium chloride containing 0.01 M disodium hydrogen phosphate and 0.2 mM EDTA (pH 7.4). Fractions (0.5 ml) were collected (0.5 ml/min) and total cholesterol and TG levels were determined enzymatically as described above.

### Ultracentrifugation

Lipoprotein fractions from pooled plasma samples were separated by buoyant density ultracentrifugation according to established methods (28). Briefly, 100 μl of pooled plasma was loaded into micro-ultracentrifuge tubes (Beckman). The samples were centrifuged for 12 h in a 42.2 Ti rotor at 175,000 *g* at 18°C (Beckman). The top 50 μl fraction containing VLDL and chylomicron remnants (δ < 1.006 g/ml) was collected and used for analysis.

### Western blot analysis

Liver (25 μg), brown adipose tissue (BAT) (20 μg), and gonadal WAT (gWAT) (20 μg) homogenized in PBS, 2 mg/ml BSA, and 5 U/ml heparin and isolated TRLs (5 μg) were analyzed by SDS-PAGE on 4–12% Bis-Tris gradient gels (NuPage; Invitrogen) with an equal amount of protein loading. Proteins were visualized by silver staining (Pierce) or after transfer to Immobilon-FL PVDF membrane (Millipore). Membranes were blocked with Odyssey blocking buffer (LI-COR Biosciences) for 30 min and incubated overnight at 4°C with respective antibodies. Goat, mouse, and rabbit antibodies were incubated with secondary Odyssey IR dye antibodies (1:14,000) and visualized with an Odyssey IR imaging system (LI-COR Biosciences). Western blot primary antibodies included: mouse anti-mouse β-actin (Sigma, A2228; 1:5,000), rabbit anti-mouse apoB (Abcam, ab20737; 1:1,000), rabbit anti-mouse apoC-III (IONIS Pharmaceuticals; 1:2,000) (29), rabbit anti-mouse apoE (Meridian Life Sciences, K23100R; 1:1,000), and goat anti-mouse LPL (provided by S. Young, University of California, Los Angeles; 10 μg/ml).

### Postprandial clearance studies

After 5 h fasting, mice were given a 250 μl bolus of corn oil (Sigma-Aldrich) by oral gavage. At the indicated time points, mice were bled via the tail vein. TG and cholesterol levels were measured as described above.

### Hepatic VLDL-TG secretion

Mice were fasted for 5 h prior to a tail vein injection of Tyloxapol (10% solution in PBS; Sigma) at a dose of 0.5 mg/g body weight. Plasma was collected by tail bleeding at time points 1, 15, 30, 60, and 120 min after injection. Plasma TG levels were measured as described above.

### Lipid absorption

Intestinal lipid absorption was analyzed in mice treated with a control ASO or apoC-III ASO for 4 weeks. After 5 h fasting, the mice were injected via the tail vein with Tyloxapol (10% solution in PBS; Sigma) at a dose of 0.5 mg/g body weight followed by a 250 μl bolus of corn oil (Sigma-Aldrich) by oral gavage. Blood was drawn via the tail vein at the indicated time points and plasma TG levels were measured as described above.

### Retinyl ester excursion

Clearance of chylomicrons derived from dietary TG was measured by vitamin A excursion essentially as described (5). Briefly, 250 μl of corn oil containing 5 μCi of [11,12-^3^H]retinol (Perkin Elmer; 44 Ci/mmol) in ethanol were administered by oral gavage to mice fasted from 4:00 AM to 9:00 AM. Blood was obtained every 2 h via the tail vein at the indicated time points and [^3^H] counts in plasma were measured by liquid scintillation counting.

### Clearance of [^3^H]TRLs in vivo

Mice were treated for 4 weeks with a control ASO or apoC-III ASO. Mice were fasted for 5 h and subjected to oral gavage (250 μl/mouse) with 5 μCi [11,12-^3^H]retinol in corn oil (Sigma-Aldrich). Blood was collected 3 h post-gavage by cardiac puncture post-euthanasia using isoflurane. [^3^H]TRLs were isolated by buoyant density ultracentrifugation as described above. In parallel, an acceptor group of mice were treated with apoC-III ASO for 4 weeks, fasted for 5 h, and then injected intravenously with freshly purified [^3^H]TRLs, either enriched or depleted in apoC-III (20,000 cpm per mouse). Serial tail vein blood samples were taken at the indicated times. Radioactivity in serial plasma samples was determined by liquid scintillation counting and expressed relative to the number of counts in the circulation 1 min after injection.

### Liposyn [^3^H]triolein tissue uptake

Preparation of liposyn solution (Sigma-Aldrich) containing 5 μCi [^3^H]triolein was performed as described (5). Briefly, 10 μl of [^3^H]triolein were slowly evaporated to dryness under N_2_ in a glass vial. Five hundred microliters of 5% Liposyn solution were added and sonicated three times for 20 s at 40 W to incorporate [^3^H]triolein into the emulsion (on ice). After centrifugation (14,000 *g* for 15 min at 4°C), 100 μl of [^3^H]triolein-labeled liposyn particles were injected intravenously into mice treated for 4 weeks with a control ASO or apoC-III ASO and fasted for 5 h. Five minutes after injection, blood was collected, the mice were perfused with 10 ml PBS via the left ventricle, and the indicated tissues were harvested. The tissues were homogenized in 1 ml SOLVABLE (Perkin Elmer) at 50°C until digested. [^3^H]triolein tissue uptake and remaining [^3^H] counts in plasma were measured by liquid scintillation counting, and protein was determined by BCA assay (Thermo Fisher Scientific).

### [^3^H]FA uptake in vivo

The uptake of radiolabeled FA into various tissues was determined in mice on control ASO or apoC-III ASO. Fasted mice (5 h) were injected retro-orbitally with 1 μCi of [^3^H]palmitic acid complexed with FA-free BSA (1:1 molar ratio). The uptake of FA into tissues was measured by scintillation counting as described above.

### Binding and uptake of [^3^H]TRLs in vitro

ApoC-III-depleted and apoC-III-enriched [^3^H]TRL particles were isolated from control ASO- and apoC-III ASO-treated *Apoe*^−/−^*Ndst1*^f/f^*Alb-Cre*^+^ mice, respectively, as described above. Next, primary hepatocytes were isolated from control ASO- or apoC-III ASO-treated *Apoe*^−/−^*Ndst1*^f/f^*Alb-Cre*^+^ mice by perfusion of the liver with EDTA to dissociate the cells, followed by Percoll centrifugation as described (30). Hepatocytes were cultured in DMEM containing 10% FBS, 100 units/ml penicillin, and 0.1 mg/ml streptomycin for 48 h prior to in vitro experiments. Uptake experiments were performed in hepatocytes seeded into collagen-coated 6-well plates (Nalgene Nunc International, Pennfield, NY) at 500,000 cells/well. Sixteen hours prior to the experiment, hepatocytes were cultivated in DMEM containing 10% lipoprotein-deficient serum. On the next day, the cells were washed with PBS and purified [^3^H]TRLs were added in a concentration of 10, 20, 50, and 100 μg/ml in DMEM. After a 4 h incubation at 37°C, the cells were washed four times with PBS. Hepatocytes were solubilized in 1 M NaOH containing 1 g/l SDS. Finally, total radioactivity and total cell protein content of the lysate were determined. All uptake data were obtained as triplicates. To analyze the impact of apoE on binding and uptake, hepatocytes were reconstituted with apoE as described previously (9). In brief, purified apoE-deficient [^3^H]TRL particles isolated from *Apoe*^−/−^*Ndst1*^f/f^*Alb-Cre*^+^ mice (100 μg) were incubated with recombinant human APOE2 or -E3 (50 μg; PeproTech, Inc.), respectively, at 37°C for 1 h in PBS. ApoE-reconstituted [^3^H]TRLs were floated by ultracentrifugation and the top fraction (δ < 1.006 g/ml) was collected. Incorporation of recombinant human apoE was verified via SDS-PAGE with silver staining. Hepatocytes were cultured 48 h prior to the experiments, as described above, and reconstituted [^3^H]TRLs were added at the indicated concentrations. Binding studies were performed in a similar manner. Hepatocytes were incubated for 1 h on ice with ice-cold medium containing [^3^H]TRLs with or without lipoprotein-deficient serum, and bound [^3^H]TRLs were analyzed as described previously (9).

### [^14^C]FA uptake in vitro

Hepatocytes enriched or depleted in apoC-III were isolated from control ASO- and apoC-III ASO-treated *Apoe*^−/−^*Ndst1*^f/f^*Alb-Cre*^+^ mice, respectively, and cultured as described above. After washing with PBS, hepatocytes were incubated with 0.5 μCi [^14^C]oleic acid complexed with FA-free BSA (1:1 molar ratio) at 37°C. At the indicated time points, the cells were lysed and uptake of radiolabeled FA was determined as described above.

### Heparin push

Release of LPL into circulation was induced by heparin injections. Mice were fasted for 5 h and 50 U of heparin were injected intravenously. For postprandial studies, mice were given an oral corn oil gavage (250 μl) after 5 h fasting. Three hours later, 50 U of heparin were injected intravenously; 40 μl of blood was drawn before and 10 min after the injections and immediately chilled on ice and centrifuged (2,000 *g* for 5 min) at 4°C to prevent ongoing lipolysis. Fasted and postprandial TG levels were measured as described above.

### Lipase activity assays

Lipoprotein and hepatic lipase (HL) were determined in *Apoe*^−/−^*Ndst1*^f/f^*Alb-Cre*^+^ mice administered control ASO or apoC-III ASO for 4 weeks as described previously (31). Briefly, 20 μl of post-heparin plasma was incubated with a 100 μl [^3^H]triolein-radiolabeled emulsion for 30 min at 37°C (5). The generated FAs were extracted and the radioactivity was determined by liquid scintillation counting. The contribution of HL was determined by including 1 M NaCl in the assay, and the values were subtracted from the total lipase activity to estimate the activity attributed to LPL. To measure lipase activities in tissues, samples were minced and reconstituted in 0.6 ml PBS containing 2 mg/ml BSA and 5 U/ml heparin. Protein concentration was measured and corrected for the addition of BSA. The aim of the LPL assay in tissues was to estimate the fraction of LPL that could be released by heparin. Minced tissues were incubated for 1 h in a 37°C shaker and subsequently centrifuged at 1,000 *g* for 15 min. One hundred microliters of the supernatant were used for the lipase assay in combination with 100 μl of [^3^H]triolein-radiolabeled emulsion. The LPL activity measurements were normalized for the amount of protein.

### Clinical study

Study design and oversight of the phase 2 randomized double-blind placebo-controlled dose-ranging study designed to evaluate the pharmacodynamic effects of ISIS 304801 on fasting APOC-III and TG levels in adult patients with severe or uncontrolled hypertriglyceridemia was described previously (21). Patients assigned to the ISIS 304801 monotherapy cohort were randomly assigned in a 1:1:1 ratio to receive a weekly dose of 100, 200, or 300 mg; the patients in these dose groups were then randomly assigned in a 3:1 ratio to receive active agent or placebo. Patients assigned to the ISIS 304801-fibrate cohort were randomly assigned in a 1:1 ratio to receive a dose of 200 or 300 mg; patients in these dose groups were then randomly assigned in a 2:1 ratio to receive active agent or placebo. The study drug was administered as a single subcutaneous injection once a week for 13 weeks as monotherapy or as an add-on to fibrate treatment. The primary outcome was the percentage change in fasting total APOC3 levels from baseline (level at day −8) to the end of treatment (mean of the levels at day 85 and day 92). Lipid measurements and APOE genotypes were assessed as described (21).

### RNA sequencing

RNA was isolated as described above. RNA sequencing was performed at the Institute for Genomic Medicine at University of California, San Diego on an Illumina HiSeq 4000 using single-end read sequencing and a ribo-depleted RNA stranded library. The spliced read aligner STAR (32) was used to align sequencing reads to the mouse GRCm38 genome. Gene-level read counts were obtained with feature Counts (33) and Ensembl gene annotation. DESeq2 (34) was used to calculate differential gene expression based on uniquely aligned reads, and *P*-values were adjusted for multiple hypothesis testing with the Benjamini-Hochberg method.

### [^13^C] tracing experiments

U-[^13^C_16_]palmitic acid tracer was purchased from Cambridge Isotopes Inc. Stable isotope labeling of intracellular metabolites was performed in primary hepatocytes isolated from *Apoe*^−/−^*Ndst1*^f/f^*Alb-Cre*^+^ mice on control ASO or apoC-III ASO as described above. Tracing experiments were performed in hepatocytes seeded into collagen-coated 12-well plates (Nalgene Nunc International) at 250,000 cells/well. For analysis of FA incorporation into the TCA cycle, hepatocytes were incubated for 1 h or 4 h with serum-free DMEM supplemented with 20 μM U-[^13^C_16_]palmitic acid conjugated in a 3:1 ratio with BSA. FA biosynthesis and TCA cycle incorporation were analyzed by GC/MS analysis.

### GC/MS analysis

Polar metabolites and FAs were extracted using methanol/water/chloroform and analyzed as previously described (35). Briefly, hepatocytes were washed with saline (0.9%) after incubation with U-[^13^C_16_]palmitic acid. Ice-cold methanol and water containing norvaline as internal standard were added to collect the cells. Next, chloroform containing U-[^2^H_31_]palmitate standard was added, and the aqueous/organic layers were separated by centrifugation. Polar metabolites were derivatized in 20 μl of 2% (w/v) methoxyamine hydrochloride (Thermo Scientific) in pyridine and incubated at 37°C for 60–90 min. Samples were then silylated with 30 μl of *N*-tert-butyldimethylsilyl-*N*-methyltrifluoroacetamide with 1% tert-butyldimethylchlorosilane (Regis Technologies) at 37°C for 30–45 min. Samples were centrifuged at 21,000 *g* for 5 min and the supernatant was transferred to GC sample vials for analysis. Derivatized polar metabolites were analyzed by GC/MS using a DB-35MS column (30 m × 0.25 mm inner diameter × 0.25 μm; Agilent J&W Scientific) installed in an Agilent 7890B gas chromatograph interfaced with an Agilent 5977A mass spectrometer. Extracted nonpolar metabolites were evaporated, saponified, and esterified to form FA methyl esters (FAMEs) through addition of 500 μl of 2% (w/v) H_2_SO_4_ in methanol and incubation at 50°C for 90–120 min. FAMEs were extracted after addition of 100 μl of saturated NaCl solution with two 500 μl hexane washes and evaporated to dryness before resuspension in 50–100 μl of hexane and transfer to glass GC vials for analysis. Derivatized FAMEs were analyzed by GC/MS using a Select FAME column (100 m × 0.25 mm inner diameter × 0.25 μm; Agilent J&W Scientific) installed in an Agilent 7890A gas chromatograph interfaced with an Agilent 5975C mass spectrometer.

### Lipoprotein subclasses

Concentrations of plasma lipoprotein subclasses were measured by specific particle-size intervals from HDL to VLDL using ion mobility (36). Particle concentrations (in nanomoles per liter) were measured for lipoprotein subclasses defined by the following size intervals: HDL3/2a (76.5–105.0 Å), HDL2b (105.0–145.0 Å), midzone between HDL and LDL (145.0–180.0 Å), LDL4c (180.0–190.0 Å), LDL4b (190.0–199.0 Å), LDL4a (199.0–204.9 Å), LDL3b (204.9–208.2 Å), LDL3a (208.2–214.1 Å), LDL2b (214.1–220.0 Å), LDL2a (222.0–224.6 Å), LDL1 (224.6–233.3 Å), IDL2 (233.3–250.0 Å), IDL1 (250.0–296.0 Å), small VLDL (296.0–335.0 Å), intermediate VLDL (335.0–424.0 Å), and large VLDL (424.0–547.0 Å). LDL peak diameter was determined by ion mobility as described previously (37).

### Statistics

Statistical analyses were performed using Prism software (version 5; GraphPad Software). Data were analyzed by Wilcoxon rank-sum test, two-tailed Student’s *t*-test, or two-way ANOVA depending on normality of the data and presented as mean ± SEM. *P*-values less than 0.05 were considered significant.

## RESULTS

### ApoC-III ASO reduces plasma TG levels in *Apoe*^−/−^*Ndst1*^f/f^*Alb-Cre*^+^ mice

**Fig. 1.**
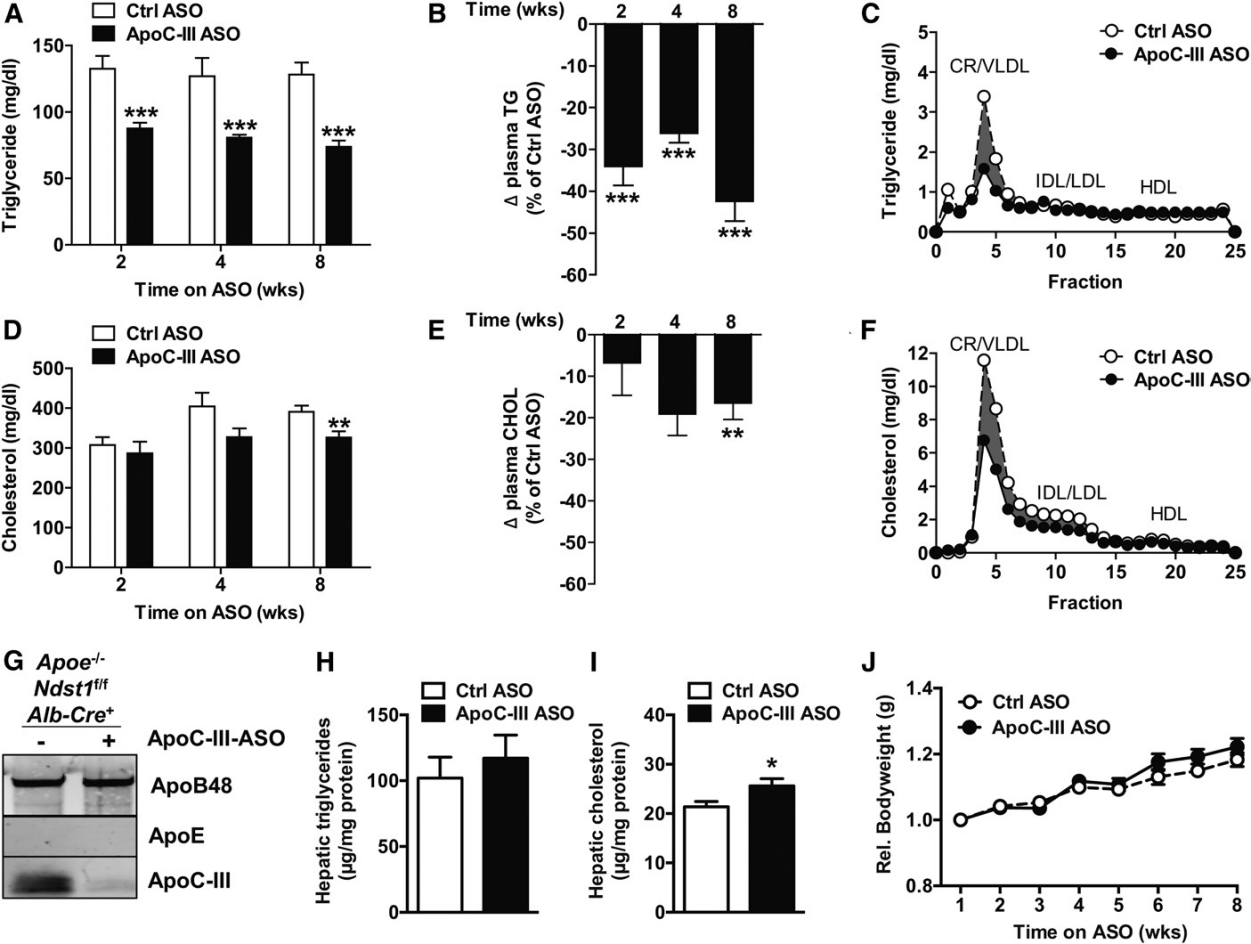
Targeting *ApoC3* with ASOs in chow-fed *Apoe*^−/−^*Ndst1*^f/f^*Alb*-*Cre*^+^ mice. A: *Apoe*^−/−^*Ndst1*^f/f^*Alb*-*Cre*^+^ mice were administered once weekly with apoC-III ASO or control (Ctrl) ASO (50 mg/kg bodyweight) for a period of 8 weeks. Fasting plasma TG levels were measured at the indicated time points. B: ApoC-III ASO-mediated relative change of plasma TG levels compared with control ASO. C: Pooled plasma samples after 4 weeks of ASO treatment were analyzed by size-exclusion FPLC, and TGs in each fraction were measured. Fasting plasma cholesterol levels after apoC-III ASO treatment (D) and relative change in cholesterol compared with control ASO (E). F: FPLC analysis of pooled plasma samples after 4 weeks of ASO treatment followed by cholesterol determination in each fraction. G: VLDLs were isolated by ultracentrifugation and pooled VLDL samples (5 μg of protein per lane) were analyzed by Western blotting with antibodies against apoB, apoC-III, and apoE. Hepatic TG (H) and cholesterol (I) levels as well as relative body weight gain (J) were measured (n = 14–17 per group, values represent mean ± SEM; *P < 0.05, **P < 0.01, ***P < 0.001).

To determine the impact of apoE on apoC-III-mediated inhibition of LDLR/LRP1-mediated TRL clearance, we administered an ASO targeting *Apoc3* (50 mg/kg/week) to chow-fed mice deficient in *Apoe* and *Ndst1* (*Apoe*^−/−^*Ndst1*^f/f^*Alb-Cre*^+^). By using *Apoe*-deficient mice compounded with hepatic *Ndst1* inactivation, we established a model to evaluate the role of apoE, a shared ligand between LDLR and LRP1, on apoC-III-mediated inhibition of TRL clearance without interference of SDC1-mediated TRL clearance. Hepatic *Apoc3* mRNA levels decreased by 88.0 ± 7.5% in *Apoe*^−/−^*Ndst1*^f/f^*Alb-Cre*^+^ mice treated with apoC-III ASO compared with control ASO. After 2, 4, and 8 weeks of ASO treatment, plasma TG levels were significantly reduced by 34.1 ± 13.3% (*P* < 0.0001), 36.5 ± 7.1% (*P* = 0.0008), and 42.4 ± 14.8% (*P* < 0.0001), respectively, compared with mice treated with control ASO (**Fig. 1A**, B). Size-exclusion chromatography analysis of plasma after 4 weeks of ASO treatment revealed that the reduction in TGs was associated with a decrease in chylomicron remnant and VLDL levels (Fig. 1C). ApoC-III ASO treatment had a minimal effect on plasma cholesterol levels (Fig. 1D–F) despite a significantly reduced apoC-III content on TRLs isolated from *Apoe*^−/−^*Ndst1*^f/f^*Alb-Cre*^+^ mice (Fig. 1G). Liver TG levels were unaffected, while hepatic cholesterol content was modestly increased by 20.1 ± 28.4% (*P* = 0.03) in the apoC-III ASO treatment group (Fig. 1H, I). Similar results were observed in *Apoe*^−/−^ mice, although less pronounced, which was likely due to compensation by apoC-III-independent TRL clearance mediated by SDC1 (supplemental Fig. S1). Hence, we used *Apoe*^−/−^*Ndst1*^f/f^*Alb-Cre*^+^ mice in subsequent experiments.

**Fig. 2.**
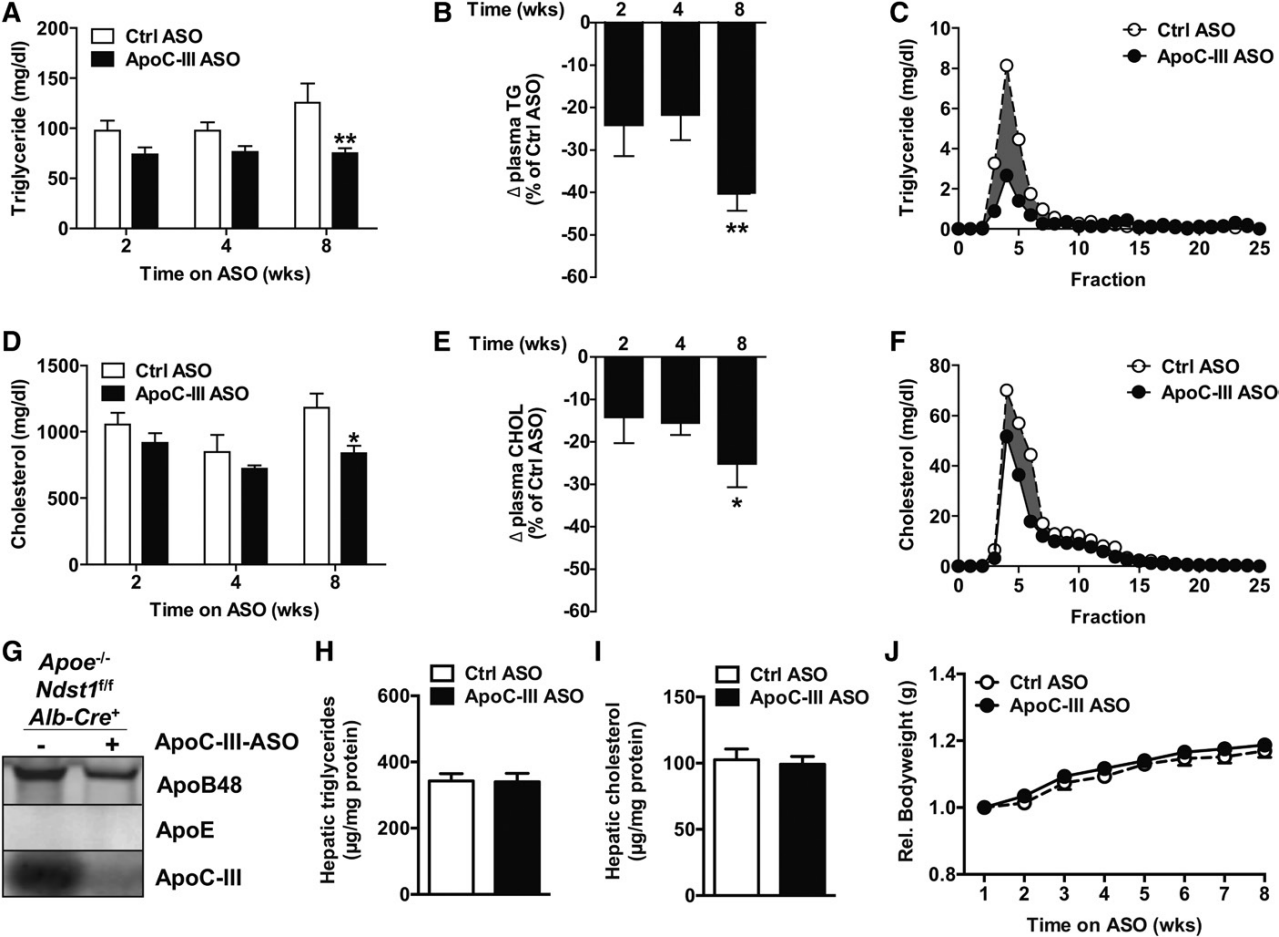
ApoC-III ASO decreases plasma TGs in *Apoe*^−/−^*Ndst1*^f/f^*Alb*-*Cre*^+^ mice on a WD. A: ApoC-III ASO or control (Ctrl) ASO (50 mg/kg bodyweight) was administered to *Apoe*^−/−^*Ndst1*^f/f^*Alb*-*Cre*^+^ mice fed a WD for 8 weeks, and fasting plasma TG values were measured after 2, 4, and 8 weeks. B: The relative change in plasma TGs upon apoC-III ASO compared with control ASO. C: Size-exclusion FPLC analysis of pooled plasma samples to determine TGs in CR/VLDL, IDL/LDL, and HDL fraction. Measurement of fasting plasma cholesterol levels after apoC-III administration (D) and relative change in cholesterol compared with control ASO (E). F: Pooled plasma samples were analyzed by FPLC and cholesterol was measured in each fraction. G: Western blot detection of apoB, apoC-III, and apoE in pooled VLDL samples (5 μg of protein per lane). Hepatic TG (H) and cholesterol (I) levels as well as relative body weight gain (J) were measured (n = 13, values represent mean ± SEM; *P < 0.05, **P < 0.01).

We next analyzed the impact of apoC-III ASO administration on *Apoe*^−/−^*Ndst1*^f/f^*Alb-Cre*^+^ mice fed a WD. In general, the WD increased cholesterol levels, as expected, and decreased TG levels, as observed in previous studies using *Apoe*^−/−^ mice (30). Interestingly, in response to apoC-III ASO treatment, no significant differences in TG and cholesterol levels were observed at 2 and 4 weeks; though again, a minimal decrease in cholesterol occurred at 8 weeks (**Fig. 2D**–F) of apoC-III ASO treatment compared with the control ASO group (Fig. 2A–F). Again, apoC-III ASO administration markedly reduced *Apoc3* expression by 92.0 ± 1.9% and apoC-III content on TRLs isolated from *Apoe*^−/−^*Ndst1*^f/f^*Alb-Cre*^+^ mice on the WD (Fig. 2G). Hepatic TG and cholesterol levels were elevated on the WD compared with chow diet but not different between treatment groups (Fig. 2H, I). No differences in bodyweight gain were observed during the treatment period on both chow and WD diet as a result of apoC-III knockdown (Figs. 1J, 2J). Taken together, the results suggest that apoC-III lowering by ASOs can improve plasma TG levels in the absence of *Apoe* expression.

### Postprandial TG response and VLDL production are unaltered by apoC-III ASO treatment

**Fig. 3.**
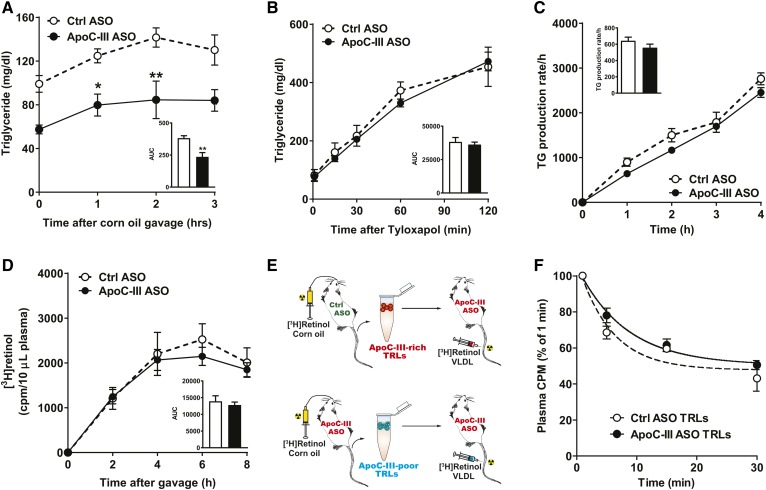
ApoC-III ASO treatment does not affect hepatic VLDL production and TRL clearance in *Apoe*^−/−^*Ndst1*^f/f^*Alb*-*Cre*^+^ mice. A: *Apoe*^−/−^*Ndst1*^f/f^*Alb*-*Cre*^+^ mice were on apoC-III ASO or control (Ctrl) ASO for 4 weeks. Fasted mice (5 h) were given a 250 μl bolus of corn oil by oral gavage to measure fat tolerance. Postprandial TG levels were measured 0, 1, 2, and 3 h after gavage (n = 3 per group). B: Hepatic VLDL production was determined after intravenous injection of Tyloxapol (0.5 mg/g bodyweight) to block lipase activity. TRL uptake and plasma TG levels were measured at the indicated time points (n = 3–5 per group). C: Intestinal lipid absorption was analyzed by intravenous injection of Tyloxapol followed by oral gavage of corn oil (250 μl). Plasma TG levels were measured at the indicated time points (n = 3). D: Hepatic TRL clearance was analyzed by retinyl ester excursion. [^3^H]retinol (5 μCi) in 250 μl corn oil was orally gavaged and the remaining counts in the plasma were determined by liquid scintillation counting after 2, 4, 6, and 8 h (n = 3). E: Schematic overview of [^3^H]TRL clearance experiments in vivo. F: Isolated [^3^H]TRLs enriched (control ASO) or depleted (apoC-III ASO) in apoC-III were injected intravenously into *Apoe*^−/−^*Ndst1*^f/f^*Alb*-*Cre*^+^ mice on apoC-III ASO (n = 2). Clearance of [^3^H]TRLs was assessed by measuring the counts remaining in the plasma relative to the counts recovered 1 min after injection (values represent mean ± SEM; *P < 0.05, **P < 0.01).

To investigate whether suppression of apoC-III improves clearance of dietary TGs independently of apoE, we performed a fat tolerance test (**Fig. 3A**). Fasted chow-fed *Apoe*^−/−^*Ndst1*^f/f^*Alb-Cre*^+^ mice were given corn oil by oral gavage and plasma TG levels were analyzed at the indicated time points. ApoC-III ASO-treated mice had lower basal and postprandial TG levels (Fig. 3A). Similar data were generated in WD-fed *Apoe*^−/−^*Ndst1*^f/f^*Alb-Cre*^+^ mice (supplemental Fig. S2). The reduction in pre- and post-prandial plasma TG levels induced by apoC-III ASO could not be explained by changes in intrahepatic VLDL secretion of TGs (Fig. 3B) or intestinal lipid absorption (Fig. 3C) as measured by accumulating plasma TG levels after intravenous injection of the lipase inhibitor, Tyloxapol, under fasting and feeding (corn oil gavage) conditions, respectively.

We previously showed that apoC-III ASO significantly improved hepatic [^3^H]retinol-TRL clearance in *Ndst1*^f/f^*Alb-Cre*^+^ mice (5). To evaluate whether apoC-III modulates hepatic TRL clearance in the absence of apoE, *Apoe*^−/−^*Ndst1*^f/f^*Alb-Cre*^+^ mice treated with control or apoC-III ASO were given an oral bolus of corn oil containing [^3^H]retinol, which is converted to retinol esters and packaged into chylomicrons. Remarkably, [^3^H]retinol excursion was similar between apoC-III ASO- and control ASO-treated *Apoe*^−/−^*Ndst1*^f/f^*Alb-Cre*^+^ mice (Fig. 3D), suggesting that apoE is crucial for apoC-III-mediated inhibition of hepatic TRL clearance. Additional support for this conclusion was obtained by measuring the clearance rate of apoC-III-rich and apoC-III-poor [^3^H]TRL particles in vivo. *Apoe*^−/−^*Ndst1*^f/f^*Alb-Cre*^+^ mice treated with apoC-III ASO or control ASO were given [^3^H]retinol-radiolabeled corn oil by gavage to generate [^3^H]retinol-labeled apoC-III-depleted and apoC-III-enriched [^3^H]TRLs, respectively (Fig. 3E). Equal counts of apoC-III-enriched or apoC-III-depleted murine [^3^H]TRLs (20,000 cpm per mouse) were injected into recipient *Apoe*^−/−^*Ndst1*^f/f^*Alb-Cre*^+^ mice. All recipient mice had been pretreated with apoC-III ASO for 4 weeks to minimize the association of endogenously produced apoC-III with the injected [^3^H]TRLs (Fig. 3E). Unlike previous results in *Ndst1*^f/f^*Alb-Cre*^+^ mice (5), the clearance rates of apoC-III-enriched or apoC-III-depleted [^3^H]TRLs injected into *Apoe*^−/−^*Ndst1*^f/f^*Alb-Cre*^+^ mice were identical (Fig. 3F). [^3^H]TRL tissue distribution in *Apoe*^−/−^*Ndst1*^f/f^*Alb-Cre*^+^ mice was unaffected by the presence or absence of apoC-III on [^3^H]TRLs (data not shown). Together, the findings support that lowering of plasma TG levels by apoC-III ASOs in the absence of apoE expression is not a result of improved hepatic TRL clearance or altered VLDL and chylomicron production.

### ApoE is required for apoC-III-mediated inhibition of hepatic TRL clearance

**Fig. 4.**
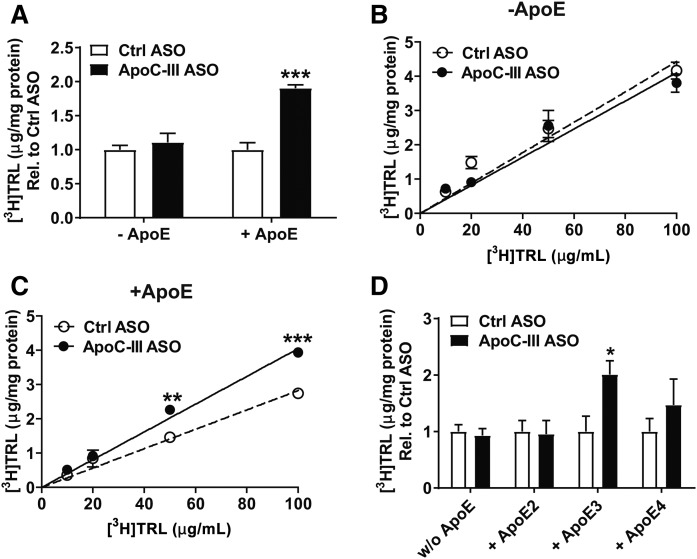
ApoC-III inhibition of TRL clearance requires apoE. A–D: Primary hepatocytes were isolated from *Apoe*^−/−^*Ndst1*^f/f^*Alb*-*Cre*^+^ mice and binding (4°C) and uptake (37°C) of [^3^H]retinol-labeled apoC-III-enriched and apoC-III-deficient TRL particles were analyzed. A: Binding of [^3^H]TRLs (100 μg/ml) enriched or deficient in apoC-III was determined after incubation in primary hepatocytes in the absence or presence of apoE at 4°C for 1 h. Binding and uptake was assessed after incubation of [^3^H]retinol-labeled TRLs at 37°C for 4 h in a dose-dependent manner (10/20/50/100 μg/ml) in the absence (B) and presence (C) of apoE. D: Reconstituted [^3^H]TRLs (50 μg) were evaluated in their binding and uptake capacity in primary hepatocytes (n = 3 per condition in all experiments, values represent mean ± SEM; *P < 0.05, **P < 0.01, ***P < 0.001).

We further analyzed the importance of apoE in apoC-III-mediated hindrance of hepatic TRL clearance. [^3^H]retinol-radiolabeled apoC-III-depleted and apoC-III-enriched TRLs (Fig. 3E) were evaluated for their binding and uptake capacity by primary hepatocytes isolated from apoC-III ASO-treated *Apoe*^−/−^*Ndst1*^f/f^*Alb-Cre*^+^ mice. Binding (4°C) and uptake (37°C) of apoE-deficient [^3^H]retinol-labeled TRLs were not affected by the presence or absence of apoC-III (**Fig. 4A**). In contrast, reconstitution of the same [^3^H]retinol-labeled apoE-deficient TRLs with apoE resulted in a significant reduction in clearance of apoC-III-enriched TRLs compared with apoC-III-depleted TRLs (Fig. 4A). Incubating primary hepatocytes with increasing concentrations of [^3^H]TRLs revealed a dose-dependent inhibition of TRL clearance by apoC-III in the presence but not in the absence of apoE (Fig. 4B, C). Human APOE exists in three isoforms (APOE2, APOE3, and APOE4) with different affinity for LDLR and LRP1. APOE4 and APOE3 having relatively equal affinity for LDLR and LRP1 compared with apoE2, which has impaired LDLR (<2% of apoE3 affinity for LDLR) and LRP1 (30% of apoE3 of apoE3 affinity for LRP1) binding (11, 38). Hence, we evaluated the binding and uptake of [^3^H]TRLs reconstituted with different human APOE isoforms in primary hepatocytes isolated from apoC-III ASO-treated *Apoe*^−/−^*Ndst1*^f/f^*Alb-Cre*^+^ mice. Reconstitution of apoC-III-depleted and apoC-III-enriched TRLs with human recombinant apoE isoforms was detected as a 35 kDa band (supplemental Fig. S3). Murine [^3^H]TRLs bearing human recombinant APOE3 showed improved binding and uptake when apoC-III was depleted (Fig. 4D). The addition of APOE4 resulted in a 1.5-fold increase in uptake of apoC-III-depleted TRLs compared with the apoC-III-enriched TRLs (Fig. 4D). In contrast, apoC-III did not affect binding and uptake of [^3^H]TRLs reconstituted with the APOE2 isoform (Fig. 4D) (11, 39). The results show that apoC-III can only inhibit hepatic TRL clearance in the presence of APOE isoforms that can functionally bind LDLR and LRP1.

### ApoC-III affects FA partitioning to liver and adipose tissue in apoE-deficient mice

**Fig. 5.**
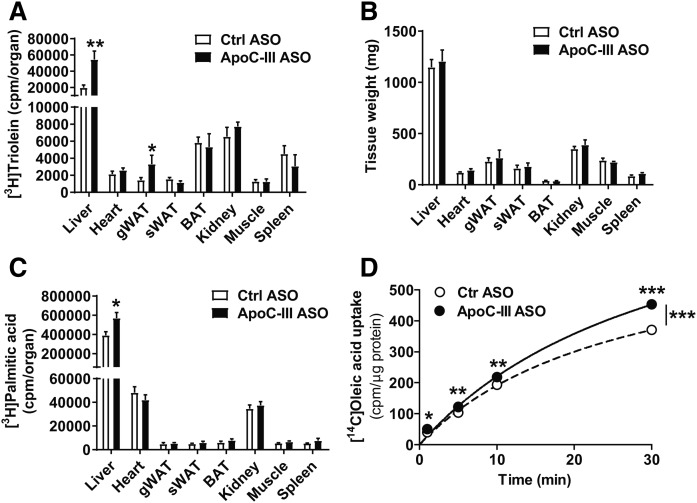
ApoC-III lowering increased FA uptake in liver and WAT in *Apoe*^−/−^*Ndst1*^f/f^*Alb*-*Cre*^+^ mice. A: *Apoe*^−/−^*Ndst1*^f/f^*Alb*-*Cre*^+^ mice were fasted for 5 h. After a 5 min intravenous injection of [^3^H]triolein-labeled liposyn particles (100 μl), blood and the indicated tissues were harvested (A) and (B) tissue weights were determined (B). [^3^H]triolein tissue uptake was determined of the homogenized tissues by liquid scintillation counting (n = 4–6). C: [^3^H]palmitic acid uptake into various tissues of control (Ctrl) ASO- and apoC-III ASO-treated mice was analyzed by retro-orbital injection of 1 μCi (n = 4–6). D: Primary hepatocytes were isolated from mice administered with control ASO or apoC-III ASO and binding and uptake of [^14^C]oleic acid was assessed in vitro. Hepatocytes were incubated with 0.5 μCi of radiolabeled FAs at 37°C, and incorporation was measured at the indicated time points (n = 3 per condition; values represent mean ± SEM; *P < 0.05, **P < 0.01, ***P < 0.001).

**Fig. 6.**
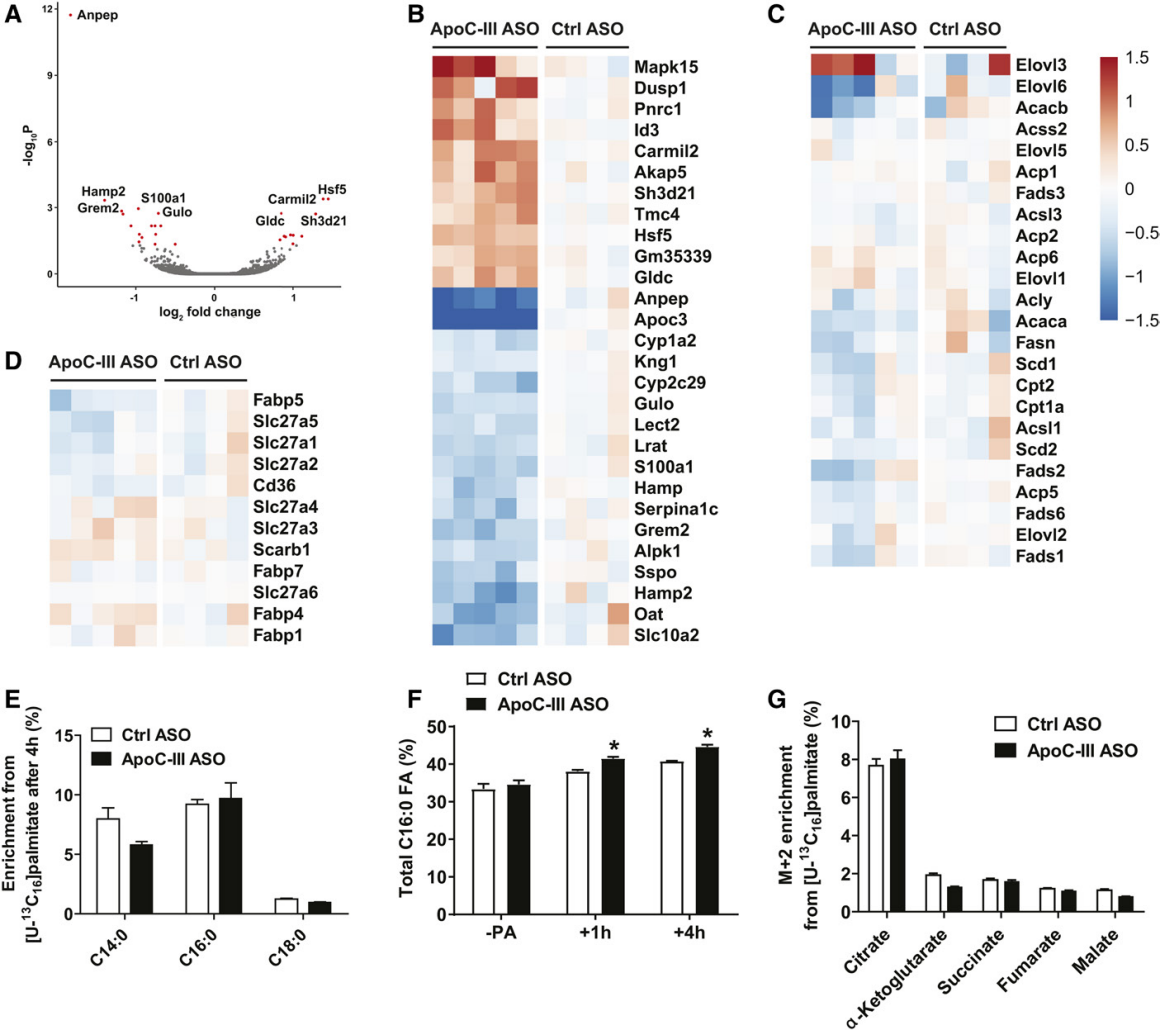
Transcriptomics and metabolomics of hepatocytes from apoC-III ASO-treated *Apoe*^−/−^*Ndst1*^f/f^*Alb*-*Cre*^+^ mice. A: Volcano-plot depicting a differential gene expression derived from RNA-seq analysis of livers isolated from fasted *Apoe*^−/−^*Ndst1*^f/f^*Alb*-*Cre*^+^ mice receiving apoC-III ASO or control (Ctrl) ASO for 8 weeks. Genes that were significantly (adjusted *P* < 0.05) up- or downregulated upon apoC-III ASO are highlighted (excluding *Apoc3*). B: A heat map showing differentially expressed protein-coding genes with adjusted *P*-value <0.05 upon apoC-III ASO (left panel, n = 5) compared with control ASO (right panel, n = 4). Heat maps of genes involved in de novo lipogenesis (C) and FA transport (D). E: ApoC-III ASO or control ASO were administered to *Apoe*^−/−^*Ndst1*^f/f^*Alb*-*Cre*^+^ mice and primary hepatocytes were isolated and incubated with 20 μM U-[^13^C_16_]palmitic acid tracer for 1 h and 4 h at 37°C for cellular FA metabolism analysis. After lipid extraction and GC/MS, enrichment of FA from U-[^13^C_16_]palmitic acid tracer and total FA composition were determined. F: Total percentage of C16:0 is shown. G: Enrichment of U-[^13^C_16_]palmitic acid tracer in metabolites of the TCA cycle (n = 3–4 per condition, values represent mean ± SEM; *P < 0.05, **P < 0.01, ***P < 0.001).

Initially, apoC-III was shown to be an inhibitor of LPL activity (40, 41). As we did not find any alterations in hepatic TRL clearance and VLDL production, we tested the possibility that apoC-III primarily affects LPL activity when apoE expression is absent by injecting reconstituted Liposyn TRL particles radiolabeled with [^3^H]triolein into *Apoe*^−/−^*Ndst1*^f/f^*Alb-Cre*^+^ mice. Five minutes after intravenous injection of [^3^H]triolein Liposyn TRLs, we analyzed tissue distribution of TRL-derived FAs. In *Apoe*^−/−^*Ndst1*^f/f^*Alb-Cre*^+^ mice, apoC-III ASO treatment was associated with a significant 2.8-fold and 2.3-fold increase in radiolabeled FA partitioning to the liver and gWAT, respectively (**Fig. 5A**). Plasma [^3^H]triolein levels trended lower in the apoC-III ASO-treated mice (supplemental Fig. S4). ApoC-III ASO treatment did not affect FA distribution to subcutaneous WAT, kidney, spleen, and oxidative tissues, such as heart, BAT, and skeletal muscle. The apoC-III ASO treatment did not affect tissue weights (Fig. 5B). In a parallel experiment, we injected BSA-associated [^3^H]palmitic acid (16:0) intravenously and harvested organs 5 min later. ApoC-III ASO-treated *Apoe*^−/−^*Ndst1*^f/f^*Alb-Cre*^+^ mice showed increased [^3^H]palmitic acid uptake into the liver (Fig. 5C), while other tissues, including gWAT, showed a similar FA uptake compared with control ASO treatment (Fig. 5C). In agreement with the in vivo data, primary hepatocytes isolated from *Apoe*^−/−^*Ndst1*^f/f^*Alb-Cre*^+^ mice treated with apoC-III ASO showed a significant increase in [^14^C]oleic acid uptake in a time-dependent manner compared with control ASO-treated mice (Fig. 5D). Taken together, the results suggest that the metabolic fate of FA is altered by apoC-III ASO knockdown. RNA sequencing on liver samples isolated from *Apoe*^−/−^*Ndst1*^f/f^*Alb-Cre*^+^ mice revealed minimal differences in hepatic gene expression. Compared with control ASO treatment, only 11 genes were upregulated >1.5-fold and 17 genes downregulated >1.5-fold, taking adjusted *P*-values <0.05 (**Fig. 6A**, B). No differences in expression of genes involved in de novo lipogenesis (Fig. 6C), FA receptors, and transporter proteins (Fig. 6D) were observed. Similarly, intracellular processing and utilization of FAs in energy metabolism in primary hepatocytes isolated from *Apoe*^−/−^*Ndst1*^f/f^*Alb-Cre*^+^ mice treated with control or apoC-III ASO were unaltered, notwithstanding increased FA uptake upon apoC-III ASO administration (Fig. 6E, F). Taken together, the results suggest that apoC-III ASO treatment increased FA uptake into gWAT and liver in *Apoe*^−/−^*Ndst1*^f/f^*Alb-Cre*^+^ mice.

### ApoC-III inhibits LPL activity in adipose tissue from apoE-deficient mice

**Fig. 7.**
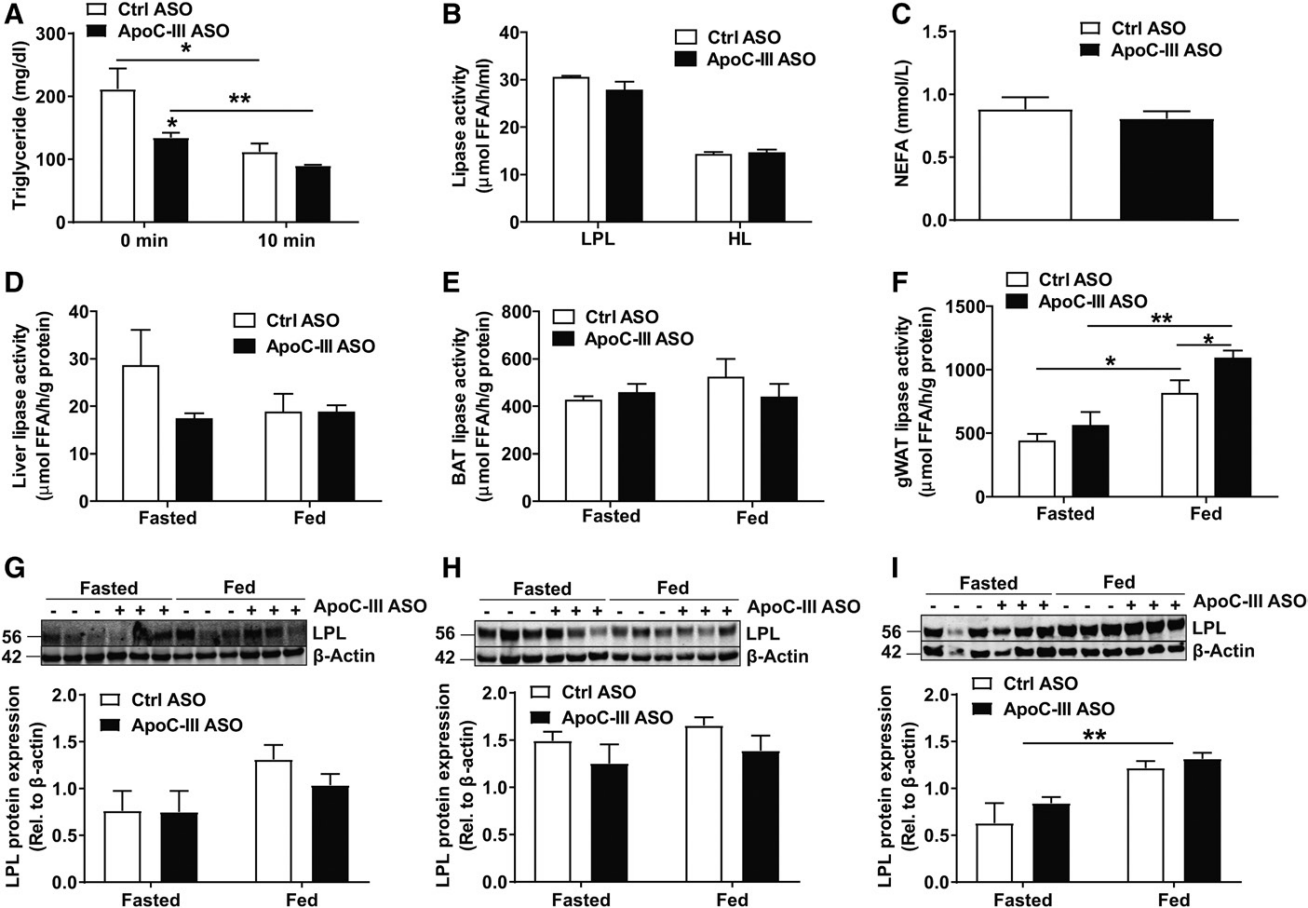
ApoC-III inhibits lipase activity in WAT from *Apoe*^−/−^*Ndst1*^f/f^*Alb*-*Cre*^+^ mice. A: Plasma TG levels were measured 3 h after an oral corn oil gavage (250 μl) before and 10 min after an intravenous heparin injection (50 U per mouse) (n = 3). B: Lipoprotein and HL activities were analyzed in post-heparin plasma in mice administered with control (Ctrl) ASO or apoC-III ASO (n = 5–6). C: Fasting NEFA values in mice treated with ASOs for 4 weeks (n = 6–8). D–F: Lipase activity was determined in liver (D), BAT (E), and gWAT (F) isolated from fasted (5 h) or fed *Apoe*^−/−^*Ndst1*^f/f^*Alb*-*Cre*^+^ mice (n = 4–6). Detection of LPL in liver (25 μg) (G), BAT (20 μg) (H), and gWAT (20 μg) (I) by Western blotting. LPL expression was quantified relative to β-actin (n = 3 per group, values represent mean ± SEM; *P < 0.05, **P < 0.01).

The increased distribution of TRL-derived FA to liver and gWAT in apoC-III-treated *Apoe*^−/−^*Ndst1*^f/f^*Alb-Cre*^+^ mice suggests increased LPL activity. To test this hypothesis, *Apoe*^−/−^*Ndst1*^f/f^*Alb-Cre*^+^ mice treated with control or apoC-III ASO were intravenously injected with heparin to release LPL from endothelial cell surfaces 3 h after an oral gavage of corn oil to mimic the postprandial state (**Fig. 7A**). Initial postprandial TG levels (*t* = 0 min) were decreased in *Apoe*^−/−^*Ndst1*^f/f^*Alb-Cre*^+^ mice treated with apoC-III ASO compared with control ASO as expected. Heparin-induced LPL release lowered plasma TG levels to a similar level in both groups, suggesting adequate lipase activity. Unlike previous observations in apoC-III knockout and apoC-III transgenic mouse models, we did not observe an increase in pre- and post-heparin plasma lipase activity (data not shown) and plasma LPL activity and HL activity when using plasma from apoC-III ASO-treated mice compared with plasma from control ASO-treated mice (Fig. 7B). Fasting NEFA levels did not change, indicating lack of an effect on FA release by adipocytes (Fig. 7C). Heparin treatment is valuable to estimate the total amount of LPL that is attached to capillary walls. However, this measure does not account for the complex regulation of LPL activity that may be conducted by other factors like apoC-I, apoC-II, ANGPTL3, ANGPTL4, and ANGPTL8 (42, 43). These are not necessarily tissue specific or dependent on LPL being attached to the capillary walls, but their impact is not measured in the in vitro LPL activity assay. Given the increased FA distribution to gWAT and liver, we tested tissue-specific LPL activity under fasting and feeding conditions in liver, gWAT, and BAT (Fig. 7D–F) as described previously (31). Under fasting conditions, no differences in lipase activity between apoC-III-treated versus control ASO-treated *Apoe*^−/−^*Ndst1*^f/f^*Alb-Cre*^+^ mice were observed in all organs tested. In the fed state, significantly higher lipase activity and LPL protein levels were observed in gWAT compared with fasting conditions in both treatment groups (Fig. 7F). More importantly, apoC-III ASO treatment significantly increased LPL activity in gWAT isolated from fed mice by 34.5 ± 16.9% compared with the control ASO group (Fig. 7F, *P *= 0.049), while LPL protein expression in gWAT was similar between apoC-III- and control ASO-treated *Apoe*^−/−^*Ndst1*^f/f^*Alb-Cre*^+^ mice (Fig. 7I). In contrast, no differences in total heparin releasable lipase activity and LPL levels were detected in liver and BAT from fed mice (Fig. 7D, E, G, H).

**Fig. 8.**
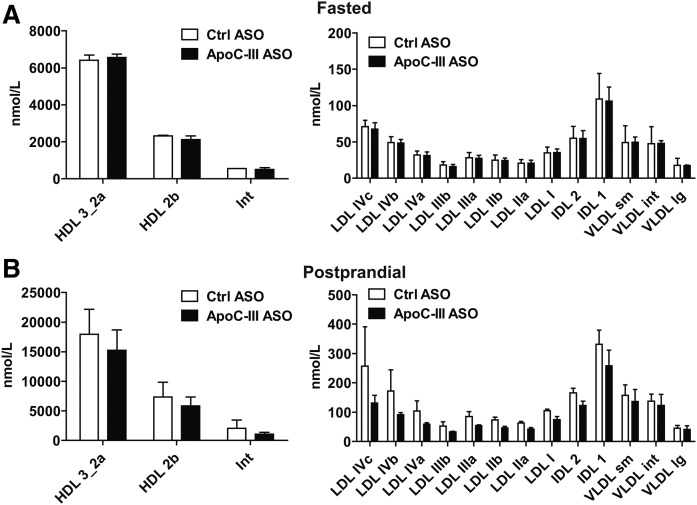
ApoC-III inhibition does not affect the size distribution of lipoprotein subclasses. Plasma samples of fasted (A) and fed (Β) *Apoe*^−/−^*Ndst1*^f/f^*Alb*-*Cre*^+^ mice on a chow diet administered with apoC-III ASO or control (Ctrl) ASO over 8 weeks were collected. Concentrations of plasma lipoprotein subclasses were measured by particle-size intervals from HDL to VLDL using ion mobility. The data represent HDL subclasses and intermediates, and show LDL, IDL, and VLDL subclasses (n = 3 pooled samples, three samples per pool, values represent mean ± SEM).

We next isolated TRLs via ultracentrifugation (δ < 1.006) to test whether the increased tissue LPL activity was associated with altered TRL composition and size upon apoC-III ASO treatment. In agreement with increased LPL activity, TRLs from apoC-III ASO-treated *Apoe*^−/−^*Ndst1*^f/f^*Alb-Cre*^+^ mice on chow diet showed a significant 63% reduction in TG levels (**Table 1**, P = 0.008). In contrast, total cholesterol levels were significantly increased (*P* = 0.048) as a result of a 1.4-fold increase in cholesterol ester (CE) content. Consequently, the TG to CE ratio was significantly reduced in TRLs from *Apoe*^−/−^*Ndst1*^f/f^*Alb-Cre*^+^ mice after administration of apoC-III ASO (Table 1, P = 0.002). The free cholesterol and protein content of the TRL particles remained unchanged and the apoC-III ASO treatment did not affect TRL particle size and concentration (**Fig. 8**). It is likely that the overall changes in TRL size and composition are too subtle to detect. Similarly, we analyzed TRLs isolated from *Apoe*^−/−^*Ndst1*^f/f^*Alb-Cre*^+^ mice on a WD. We observed a very modest reduction in TG content and no alteration in the TG to CE ratio in TRLs upon apoC-III ASO treatment, which is in line with the modest plasma TG lowering under high-fat diet feeding. The cumulative results altogether suggest that apoC-III knockdown reduces plasma TG levels in apoE-deficient mice as a result of increased LPL activity.

**TABLE 1.** ApoC-III ASO decreases the TG amount of TRL particles lacking apoE

	Chow Diet		WD	
	Control ASO	ApoC-III ASO	Control ASO	ApoC-III ASO
TGs (μmol/mg protein)	7.22 ± 1.33	2.68 ± 0.31*^a^*	7.24 ± 0.55	5.52 ± 0.23*^b^*
Cholesterol (μmol/mg protein)	1.48 ± 0.16	1.98 ± 0.15*^b^*	3.55 ± 0.19	3.94 ± 0.19
Free cholesterol (μmol/mg protein)	0.96 ± 0.13	1.09 ± 0.06	1.87 ± 0.14	2.38 ± 0.13*^b^*
Cholesteryl ester (μmol/mg protein)	0.29 ± 0.06	0.49 ± 0.06*^b^*	0.93 ± 0.04	0.87 ± 0.06
TG/CE	34.2 ± 10.1	6.17 ± 1.20*^b^*	7.86 ± 0.63	6.57 ± 0.64

TRLs were isolated from *Apoe*^−/−^*Ndst1*^f/f^*Alb*-*Cre*^+^ mice on chow diet (n = 6 per group) of WD (n = 6 per group). Values represent mean ± SEM.

a *P* < 0.01.

b *P* < 0.05.

### Volanesorsen reduces plasma TGs in patients with the APOE2/E2 allele

**Fig. 9.**
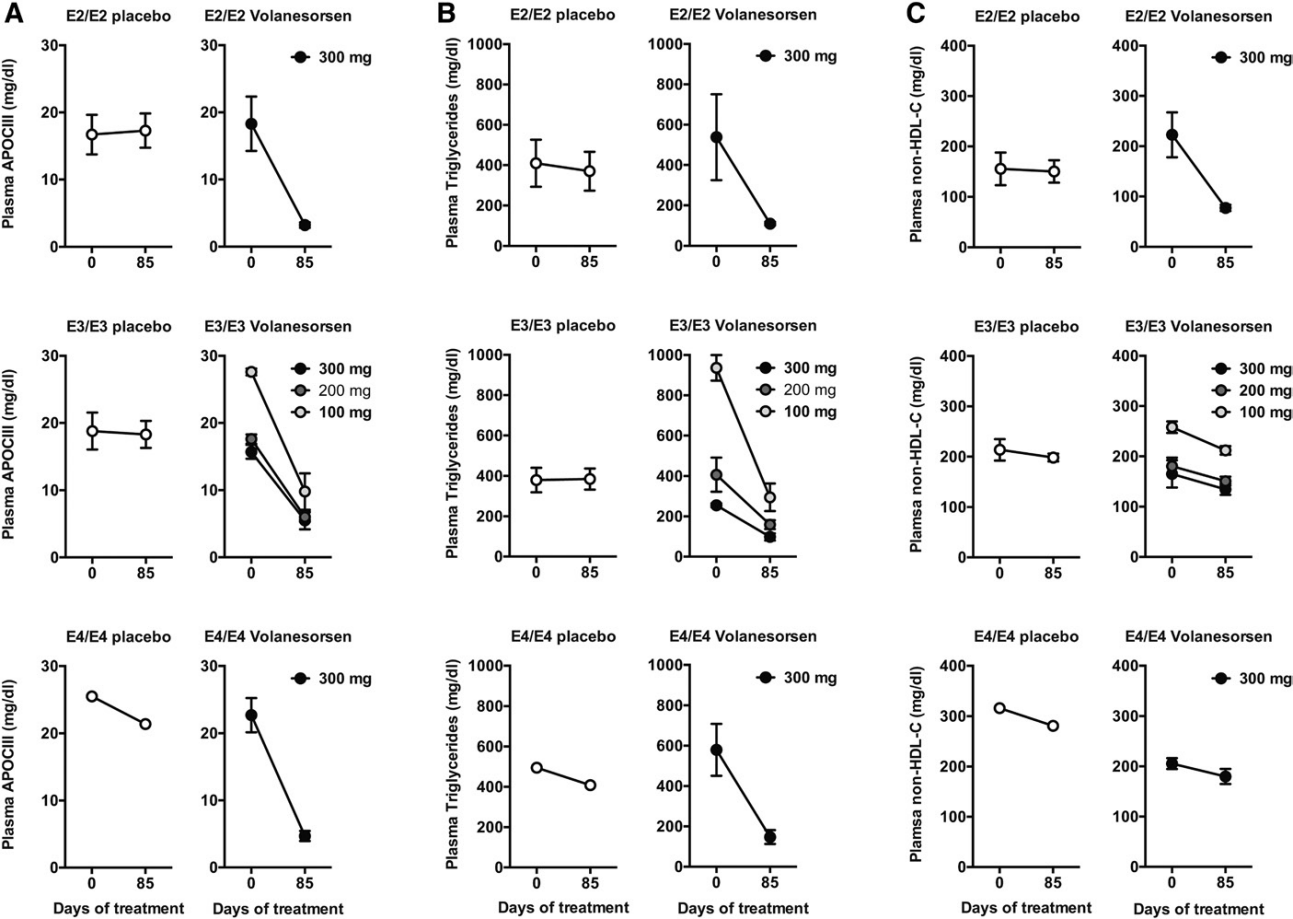
Volanesorsen lowers plasma TG levels independent of allelic *APOE* expression in a hypertriglyceridemia cohort. In a randomized double-blind placebo-controlled dose-ranging phase 2 study, placebo or volanesorsen (100–300 mg) was administered once weekly to patients with different allelic apoE background. Plasma APOC-III (A), plasma TG levels (B), and plasma non-HDL-C levels (C) were measured at baseline and after 85 days of either placebo or volanesorsen treatment in patients homozygous for APOE2 (E2/E2), APOE3 (E3/E3), and APOE4 (E4/E4), respectively (E2/E2: placebo n = 4, volanesorsen n = 3; E3/E3: placebo n = 7, volanesorsen 100 mg n = 3, 200 mg n = 7, 300 mg n = 3; E4/E4: placebo n = 1, volanesorsen n = 4; values represent mean ± SEM, data are shown for patients on monotherapy combined with fibrates).

Treatment with the human apoC-III ASO, volanesorsen, effectively lowers plasma apoC-III and TG levels in normolipidemic and hyperlipidemic human patients (29, 44, 45). However, the impact of the APOE isoforms expressed by the individuals on the therapeutic efficiency of volanesorsen is unclear. Based on our results, we anticipated no significant difference in the efficiency of TG lowering mediated by differential APOE isoform expression. We therefore examined the data of a previously published randomized double-blind placebo-controlled dose-ranging phase 2 study of the effects of volanesorsen in patients with hypertriglyceridemia according to the distribution of APOE isoforms among patients (21). As described previously, a total of 57 patients were treated with volanesorsen monotherapy (41 received volanesorsen and 16 received placebo), and 28 patients were treated with volanesorsen in combination to established fibrate therapy (20 received volanesorsen and 8 received placebo) for 85 days. APOE isoform genotype and follow-up data were available for 80 patients and the impact of volanesorsen therapy is shown by all genotypes in supplemental Tables S3–S7. In general, there was no impact of APOE genotype on the impact of TG lowering mediated by volanesorsen. To illustrate these findings, we specifically examined data here of patients who were homozygous for APOE2 (E2/E2, n = 7), APOE3 (E3/E3, n = 20), and APOE4 (E4/E4, n = 5), respectively (Table 2). The APOE3 homozygous patients received placebo (n = 7, with one patient on fibrates) or 100 mg (n = 3, no patients on fibrates), 200 mg (n = 7, with four patients on fibrates), or 300 mg (n = 3, all patients on fibrates) volanesorsen, which significantly reduced APOC-III levels (**Fig. 9A**). Plasma TG levels decreased by 72.8 ± 14.3%, 56.3 ± 14.7%, and 61.0 ± 14.0% for the 100, 200, and 300 mg doses, respectively, compared with an 8.7 ± 32.2% increase in the placebo group (Fig. 9B). APOE4 homozygote patients received 300 mg volanesorsen (n = 4) or placebo (n = 1) (with or without fibrates). Volanesorsen treatment reduced plasma APOC-III and TG levels by 78.6 ± 8.5% and 73.9 ± 6.4%, respectively, compared with only 16.2% and 17.6% in the placebo group (Fig. 9A, B). Similarly, all APOE2 homozygote patients received either placebo (n = 4) or 300 mg volanesorsen (n = 3) with or without fibrates. After 85 days of weekly treatment, plasma APOC-III and TG levels dropped by 81.6 ± 6.4% and 73.9 ± 12.7%, respectively, from baseline in the volanesorsen cohort, and increased by 7.1 ± 20.0% and decreased by 0.8 ± 30.6%, respectively, in the placebo group (Fig. 9A, B). Remarkably, volanesorsen also reduced plasma non-HDL cholesterol (HDL-C) levels by 63.7 ± 7.0% in APOE2 homozygous patients (Fig. 9C) but not in APOE3 and APOE4 homozygous patients. Overall HDL-C levels increased by 42–60% in this cohort of patients administered volanesorsen, whereas HDL-C (4.4 ± 20.0%) was unaltered in the placebo groups (**Table 2**). We conclude that volanesorsen lowers plasma TG and increases HDL-C levels in a human hypertriglyceridemia patient cohort independent of the APOE genotype.

**TABLE 2.** Combined data of lipid analysis of an APOE cohort administered with placebo or volanesorsen (Ionis 304801) as a monotherapy or add-on to fibrates

APOE Isoform	Treatment		Parameter			
			APOC-III (mg/dl)	TGs (mg/dl)	HDL-C (mg/dl)	Non-HDL-C (mg/dl)
E2/E2	Placebo (n = 4)	Pre	16.7 ± 5.9	409.5 ± 233.5	37.8 ± 11.1	155.5 ± 64.6
		Post	17.3 ± 5.1	370.1 ± 192.3	41.5 ± 9.9	150.4 ± 44.4
		Change	7.1 ± 20.0%	−0.8 ± 30.6%	13.0 ± 27.1%	1.7 ± 18.9%
	Volanesorsen 300 mg (n = 3)	Pre	18.9 ± 7.0	538.3 ± 369.5	42.3 ± 12.7	222.7 ± 77.4
		Post	3.2 ± 0.7	109.3 ± 15.9	67.0 ± 21.0	77.3 ± 10.7
		Change	−81.6 ± 6.4%	−73.9 ± 12.7%	59.6 ± 21.2%	−63.7 ± 7.0%
E3/E3	Placebo (n = 7)	Pre	18.8 ± 7.3	379.6 ± 160.3	34.0 ± 5.8	213.6 ± 55.9
		Post	18.3 ± 5.3	384.9 ± 138.6	35.5 ± 9.0	198.4 ± 20.4
		Change	4.9 ± 32.7%	8.7 ± 32.2%	4.4 ± 20.0%	−4.3 ± 14.6%
	Volanesorsen 100 mg (n = 3)	Pre	27.6 ± 0.9	936.2 ± 109.8	26.0 ± 2.0	258.0 ± 19.7
		Post	9.9 ± 4.7	249.5 ± 118.5	41.3 ± 15.5	211.8 ± 14.7
		Change	−64.6 ± 16.2%	−72.8 ± 14.3%	60.0 ± 61.1%	−17.6 ± 8.2%
	Volanesorsen 200 mg (n = 7)	Pre	17.6 ± 5.3	406.6 ± 223.7	33.0 ± 4.8	180.6 ± 44.4
		Post	6.0 ± 1.9	158.9 ± 57.9	46.8 ± 9.4	150.9 ± 23.6
		Change	−65.3 ± 10.1%	−56.3 ± 14.7%	41.9 ± 18.9%	−14.0 ± 15.2%
	Volanesorsen 300 mg (n = 3)	Pre	15.7 ± 1.8	255.2 ± 17.1	39.7 ± 14.6	165.3 ± 47.0
		Post	5.5 ± 2.3	98.0 ± 30.4	61.2 ± 29.3	134.5 ± 19.0
		Change	−64.0 ± 16.5%	−61.0 ± 14.0%	51.1 ± 44.9%	−15.5 ± 18.0%
E4/E4	Placebo (n = 1)	Pre	25.5	495.0	30.0	316.0
		Post	21.4	408.0	31.0	281.0
		Change	−16.2%	−17.6%	3.3%	−11.1%
	Volanesorsen 300 mg (n = 4)	Pre	22.7 ± 5.1	579.9 ± 257.5	26.5 ± 8.4	205.5 ± 21.6
		Post	4.7 ± 1.5	147.1 ± 68.5	39.1 ± 11.8	179.9 ± 30.1
		Change	−78.6 ± 8.5%	−73.9 ± 6.4%	48.7 ± 9.5%	−12.1 ± 15.9%

Values represent mean ± SD.

## DISCUSSION

**Fig. 10.**
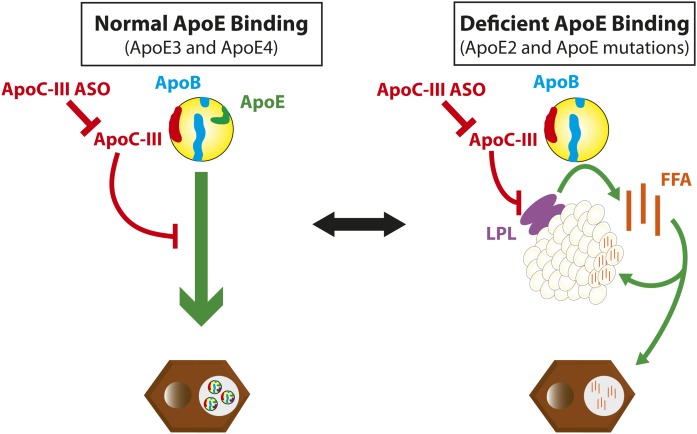
The role of apoC-III on lipoprotein metabolism varies depending on apoE. Schematic representation of how apoE determines the metabolic fate of apoC-III in TG metabolism by shifting apoC-III’s versatile role from attenuating hepatic TRL clearance to LPL inhibition.

Our results present a novel model in which apoE determines the metabolic impact of apoC-III in TG metabolism by shifting apoC-III’s action from attenuating hepatic TRL clearance to LPL inhibition (**Fig. 10**). This is supported by our observations that lowering of apoC-III levels using ASOs in the absence of apoE did not improve TRL clearance, yet significantly decreased plasma TG levels in vivo. This model is in agreement with previous studies in humans (10) and in compound deficient *Apoc3*^−/−^*Apoe*^−/−^ mice (25). Further, we show that apoC-III ASO-associated TG lowering in the absence of apoE is a result of increased lipase activity and not increased amounts of LPL on capillaries, as estimated from LPL activity in post-heparin plasma. This was not a generalized improvement in lipase activity in all LPL-target tissues, as suggested before, as we observed a localized improvement of LPL activity in WAT with subsequent augmented FA uptake into WAT and spillover of FAs into the liver.

Our data also conclusively demonstrate that apoC-III prevents hepatic TRL clearance via LDLR and LRP1 in an apoE-dependent fashion. First, ASO-mediated apoC-III reduction does not improve hepatic TRL clearance in *Apoe*^−/−^*Ndst1*^f/f^*Alb-Cre*^+^ mice, while it does improve TRL clearance in *Ndst1*^f/f^*Alb-Cre*^+^ mice (5). Finally, apoE-deficient [^3^H]retinol-radiolabeled apoC-III-depleted and apoC-III-enriched TRLs were internalized by primary hepatocytes at the same rate. Reconstitution with apoE reversed this inhibition and significantly improved hepatocyte-associated binding and uptake of apoC-III-depleted over apoC-III-enriched TRLs. Hence, we hypothesize that apoC-III prevents TRL clearance via LDLR and LRP1 by modulating apoE binding, but without affecting SDC1 interaction with apoE on TRLs. We conclude the latter from our previous observation that apoC-III did not affect TRL binding and uptake in compound LDLR- and LRP1-deficient mice, which exclusively express hepatic SDC1 to clear TRLs (5).

It remains unclear exactly how apoC-III hinders apoE-mediated TRL clearance via LDLR/LRP1 and not SDC1. Multiple mechanisms have been proposed, including direct inhibition by apoC-III of apoE-mediated TRL clearance via LDLR and LRP1 as well as competition between apoE and apoC-III for space on TRLs (13, 14, 22–24). Our observations suggest that the latter model is improbable, as apoE levels on TRLs after reconstitution were not affected by the presence or absence of apoC-III. However, previous studies reported displacement of apoE from VLDL particles when apoC-III was overexpressed or added exogenously (5, 13, 22–24, 46). Also, TRL binding and uptake after reconstitution with human APOE2 isoforms was unaffected by the presence or absence of apoC-III, providing further support that apoC-III-mediated inhibition of TRL clearance is not a result of apoE displacement. In such a displacement model, one would also expect to affect SDC1-mediated TRL clearance (9), which we never observed. A mechanism where apoC-III inhibits apoE-mediated TRL clearance independent of the apoE content on TRLs is therefore more likely. Possibilities include the concept that apoC-III inhibits TRL clearance by masking apoE and preventing or inducing a conformational change in apoE required or incompatible for efficient LDLR interactions (47, 48). Yet, such a conformation change would not affect apoE binding to SDC1 (5, 9).

The apoC-III lowering-induced reduction of plasma TGs in *Apoe*^−/−^*Ndst1*^f/f^*Alb-Cre*^+^ mice was not caused by altered VLDL production or intestinal lipid absorption (5, 25, 29, 49–52). Our observation might imply that the reported increase in lipid absorption observed in *Apoc3*^−/−^*Apoe*^−/−^ mice is a result of the loss of intestinal apoC-III expression and not related to its expression in the liver (25, 53). As neither of the previous metabolic pathways were affected, we postulated that increased lipase activity lowered TGs and increased FA uptake in *Apoe*^−/−^*Ndst1*^f/f^*Alb-Cre*^+^ mice treated with apoC-III ASO. Although our recent studies in mice did not find evidence that apoC-III modulates LPL activity (5), apoC-III has historically been established as an inhibitor of LPL (25, 40, 54). Jong et al. (25) reported that reduced plasma TGs in *Apoc3*^−/−^*Apoe*^−/−^ mice correlated with increased clearance of liposyn emulsion-associated FA and not human apoB clearance after injection of human VLDL. While this suggested increased LPL activity or expression, it was never confirmed biochemically nor was it clear if this observation was dependent on apoE expression (25). Our study significantly advances our understanding of the impact of apoC-III on lipid metabolism and provides a more intricate understanding of how apoC-III can impact LPL function. First off, we establish that in the absence of apoE, lowering apoC-III augments tissue LPL activity and not its expression, as we show that this is not a result of increasing heparin-releasable LPL levels. Second, we provide evidence suggesting that apoC-III enhances WAT LPL activity with ensuing increased free FA uptake in gWAT and liver. Interestingly, apoC-III ASO treatment was able to increase gWAT LPL activity beyond levels in tissue normally targeted by LPL upon fasting, such as skeletal muscle and heart. We do want to point out that, of course, other factors could affect LPL activity and LPL protein levels in tissue when measured in the presence of heparin. Finally, the use of human TRLs is a poor model for studying hepatic TRL clearance in mice, as human apoB-TRLs have a very weak affinity for murine LDLR. This makes apoB-clearance difficult to interpret in the study by Jong et al. (25). By using murine core [^3^H]retinol-labeled murine TRLs, we were able to more elegantly study the impact of apoC-III on TRL clearance in the absence of apoE both in vivo and in vitro. Moreover, our apoE in vitro and in vivo complementation studies clearly establish the need for apoE in order for apoC-III to affect TRL clearance. When comparing the current observation in *Apoe*^−/−^*Ndst1*^f/f^*Alb-Cre*^+^ mice to our previous data obtained in an LPL-deficient models (5), it is interesting to note that apoC-III ASO treatment increased both TRL clearance and LPL activity, respectively, in these models, resulting in a relative similar capacity to reduce plasma TG levels.

Increased LPL activity in gWAT and elevated rates of FA uptake into liver, as shown in vivo by both uptake of [^3^H]triolein-labeled liposyn particles and [^3^H]palmitic acid and in vitro by [^14^C]oleic acid and gWAT might result in altered FA metabolism and energy usage. Recently, an apoC-III gain-of-function mutation (Gln38Lys) was associated with increased hepatic de novo lipogenesis measured by upregulation of SREBP-1/2, FAS, ACC1, and CD-36, and FA synthesis (52). Another study in rats also showed an association between lower apoC-III levels and increased FA oxidation and a decrease in plasma TG levels (55). However, RNA sequencing and metabolite analysis of livers from apoC-III ASO-treated *Apoe*^−/−^*Ndst1*^f/f^*Alb-Cre*^+^ mice only revealed minimal differences compared with control ASO-treated mice. No significant changes in genes involved in FA transport and de novo lipogenesis were detected. However, one cannot rule out that apoC-III lowering affects protein levels or the transport and interaction of FA with hepatic receptors.

Our proposed metabolic switch model has clinical relevance, as apoC-III ASO administration in a clinical study lowered plasma TG levels in hypertriglyceridemia patients independent of the expressed APOE isoform. APOE exists in three isoforms in humans (APOE2, APOE3, and APOE4) that have a different affinity for LDLR and LRP1. The receptor-binding domain of apoE (residues 135–150) is enriched in basic arginine and lysine residues, which interact with acidic amino acids of the calcium-binding repeat of LDLR (39, 56). APOE3 and APOE4 carry an arginine residue at position 158, which mediates binding to LDLR (11). In contrast, APOE2 contains a cysteine residue at this position resulting in a conformational change of the receptor-binding domain and ultimately defective binding to LDLR (<2% of normal LDLR binding activity) and LRP1 (30–50% of normal LRP1 binding activity) (11, 38). APOE2 homozygosity is associated with recessive inheritance and low penetrance of type III hyperlipoproteinemia (57). We analyzed the impact of APOE genotype in hypertriglyceridemia patients from a previous study testing the efficacy of ASO-mediated APOC-III lowering with volanesorsen in relation to apoE isoforms (19, 21, 44, 45). As expected, our retrospective analysis shows that volanesorsen reduced plasma TGs in homozygous APOE3 or APOE4 (or a combinations of those isoforms) hypertriglyceridemia patients most likely by facilitating TRL clearance via LDLR and LRP1 (5). Remarkably, APOC-III lowering also strongly reduced plasma TG levels in APOE2 homozygous patients. These results are in sharp contrast to earlier observations from *APOE2*-knockin mice where compound apoC-III inactivation did not alter total plasma TG levels (58). This contradiction with our data in human APOE2 homozygous patients suggests that human APOE isoforms differentially affect TG metabolism in humans compared with mice. The limited murine *APOE2*-knockin study did not assess the impact of apoC-III deficiency on lipid absorption, VLDL production, and TRL clearance (58). It is possible that the increased intestinal lipid absorption observed in compound deficient *Apoc3*^−/−^*Apoe*^−/−^ mice also manifests in *APOE2*-knockin mice (25). If so, this increased absorption could counterbalance any improvement on LPL-driven clearance of plasma TGs due to loss of apoC-III expression. Because human APOE2 homozygous hypertriglyceridemia patients lack the ability to efficiently clear TRLs through the LDLR/LRP1 axis, our results suggest that volanesorsen can reduce plasma TG levels independent of this apoE-driven hepatic TRL clearance pathway. In humans, genetically reducing plasma APOC-III by only 50% led to enhanced conversion of plasma VLDL to LDL and lowering of VLDL-TG by 45%, without evidence for enhanced hepatic VLDL remnants and LDL removal and accumulation (59). Thus, the consequence of lowering of APOC-III by >70% by volanesorsen in the E2/E2 homozygotes seems to allow LPL to reduce plasma TGs by approximately 80%, as noted in our study. Limitations of this unique clinical study are the small sample size, especially of patients with low-abundant homozygous APOE-isoforms (E2 and E4). Despite this restriction, most of the observed plasma TG reductions upon volanesorsen treatment were profound and present in all treated patients, accentuating the robustness of our findings.

Altogether, our study shows for the first time that the apoE genotype in hypertriglyceridemia patients does not negatively affect the efficiency of volanesorsen to lower plasma TGs. Further in-depth investigation will be needed to address exactly how apoC-III can differentially affect apoE-mediated TRL clearance by LDLR/LRP1 and SDC1. More importantly, it needs to be determined whether the distinctly different TG clearance pathways modulated by APOC-III have a differential impact on CVD and longevity, which are associated with altered plasma apoC-III levels in the human population.

## Supplementary Material

Supplemental Data
