# Supplementary material for: ApoC-III ASO promotes tissue LPL activity in the absence of apoE-mediated TRL clearance
Source: J Lipid Res. 2019 May 14;60(8):1379–95. doi: 10.1194/jlr.M093740 (PMC6672034; doi:10.1194/jlr.M093740)
Supplement: Supplemental Data [file supp_60_8_1379__index.html]

ApoC-III ASO Promotes Tissue LPL Activity in Absence of ApoE-Mediated TRL Clearance — ApoC-III ASO promotes tissue LPL activity in the absence of apoE-mediated TRL clearance — Supplemental Data 

# ApoC-III ASO promotes tissue LPL activity in the absence of apoE-mediated TRL clearance

## Supplemental Data

- Supplemental Data (.pdf, 1.0 MB) - Supplemental Data file
