# Supplementary material for: ApoC-III ASO promotes tissue LPL activity in the absence of apoE-mediated TRL clearance
Source: J Lipid Res. 2019 May 14;60(8):1379–95. doi: 10.1194/jlr.M093740 (PMC6672034; doi:10.1194/jlr.M093740)
Supplement: Supplemental Data [file 10.1194_M093740_jlr.M093740-1.pdf]

ApoC-III ASO Promotes Tissue LPL Activity in Absence of ApoE-Mediated TRL Clearance

Bastian Ramms[1,2,3,4], Sohan Patel[2,3], Chelsea Nora[2], Ariane R. Pessentheiner[2,3], Max W. Chang[1], Courtney R. Green[5], Gregory J. Golden[1,3], Patrick Secrest[1], Ronald M. Krauss[5], Christian M. Metallo[5], Christopher Benner[1], Mark J. Graham[6], Joseph L. Witztum[2], Sotirios Tsimikas[2], Jeffrey D. Esko[1,3] and Philip L.S.M. Gordts[1,2,3*]

[1]Department of Cellular and Molecular Medicine, University of California, San Diego, La Jolla, CA, USA.

[2]Department of Medicine, University of California, San Diego, La Jolla, CA, USA.

[3]Glycobiology Research and Training Center, University of California San Diego, La Jolla, CA, USA.

[4]Department of Chemistry, Biochemistry I, Bielefeld University, Bielefeld, Germany.

[5]Department of Bioengineering, University of California, San Diego, La Jolla, CA, USA.

[6]Children's Hospital Oakland Research Institute, Oakland, CA, USA.

[7]Ionis Pharmaceuticals Inc., Carlsbad, CA, USA.

***Corresponding author**

Philip L.S.M. Gordts, Department of Medicine, University of California, San Diego, La Jolla, CA 92093-0687, Ph: 858/246-0994, FAX: 858/534-5611, pgordts@ucsd.edu

Running Title: ApoE determines the metabolic impact of apoC-III

SUPPLEMENTARY TABLES AND FIGURES.

SUPPLEMENTAL TABLES

Supplemental Table S1: Murine Antisense Oligonucleotide Sequences.

Ionis ID	Name	Target Gene	Sequence
ION 440726	ApoC-III ASO	apoC-III	5'-CCAGCTTTATTAGGGACAGC-3'
ION 141923	Control ASO	N.A. (scramble)	5’-CCTTCCCTGAAGGTTCCTCC-3'

Supplemental Table S2: Real-Time PCR Primers.

Gene	Forward Primer	Reverse Primer
Apoc3	5’-TGCAGGGCTACATGGAACAA-3’	5’-TCGGACTCCTGCACGCTACTT-3’
Tbp	5’-GAAGCTGCGGTACAATTCCAG-3’	5’-CCCCTTGTACCCTTCACCAAT-3’

Supplemental Table S3: Lipid analysis of a homozygote apoE cohort administered with placebo or volanesorsen (Ionis 304801) as a monotherapy.

ApoE isoform	Treatment		Parameter ApoC-III (mg/dL)	TGs (mg/dL)	HDL-C (mg/dL)	non-HDL-C (mg/dL)
**E2/E2**	**Placebo (n = 1)**	**Pre**	11.0	190.0	41.0	101.0
		**Post**	13.8	244.0	39.0	124.0
		**Change**	26.0%	28.4%	-4.9%	22.8%
	**Volanesorsen 300 mg (n = 2)**	**Pre**	22.6 ± 4.1	687 ± 374.8	40 ± 17.0	245 ± 94.8
		**Post**	3.4 ± 0.9	118.5 ± 1.4	68.8 ± 29.3	78.5 ± 14.9
		**Change**	-85.3 ± 1.5%	-79.8 ± 10.8%	71.8 ± 0.5%	-66.6 ± 6.8%
**E3/E3**	**Placebo (n = 6)**	**Pre**	20.2 ± 6.9	408.8 ± 153.8	33.5 ± 6.2	219.5 ± 58.7
		**Post**	18.8 ± 5.6	396.7 ± 148	36.4 ± 9.5	201.7 ± 20.3
		**Change**	-2.3 ± 29.0%	1.1 ± 27.6%	8.3 ± 18.7%	-5.1 ± 15.8%
	**Volanesorsen 100 mg (n = 3)**	**Pre**	27.6 ± 0.9	936.2 ± 109.8	26.0 ± 2.0	258.0 ± 19.7
		**Post**	9.9 ± 4.7	249.5 ± 118.5	41.3 ± 15.5	211.8 ± 14.7
		**Change**	-64.6 ± 16.2%	-72.8 ± 14.3%	60.0 ± 61.1%	-17.6 ± 8.2%
	**Volanesorsen 200 mg (n = 3)**	**Pre**	23.0 ± 2.2	599.2 ± 224.2	30.7 ± 5.8	170.7 ± 62.7
		**Post**	7.4 ± 0.4	202.2 ± 60.8	41.8 ± 6.8	158.2 ± 28.1
		**Change**	-67.4 ± 4.6%	-63.0 ± 18.5%	37.9 ± 19.1%	-3.5 ± 15.7%
**E4/E4**	**Placebo (n = 0)**	**Pre**	-	-	-	-
		**Post**	-	-	-	-
		**Change**	-	-	-	-
	**Volanesorsen 300 mg (n = 3)**	**Pre**	20.4 ± 2.6	462.5 ± 129.5	30.7 ± 1.2	195.7 ± 11.0
		**Post**	4.3 ± 1.6	113 ± 7.7	45 ± 1.7	178.8 ± 36.8
		**Change**	-78.1 ± 10.3%	-74.1 ± 7.8%	47 ± 10.9%	-8.7 ± 17.7%

Values represent mean ± SD.

Supplemental Table S4: Lipid analysis of a homozygote apoE cohort administered with placebo or volanesorsen (Ionis 304801) as an add-on to fibrates.

ApoE isoform	Treatment		Parameter<br>ApoC-III (mg/dL)	TGs (mg/dL)	HDL-C (mg/dL)	non-HDL-C (mg/dL)
**E2/E2**	**Fibrate + Placebo (n = 3)**	**Pre**	18.6 ± 5.4	482.7 ± 222.9	36.7 ± 13.3	173.7 ± 65.5
		**Post**	18.5 ± 5.5	412.2 ± 211.8	42.3 ± 11.9	159.2 ± 50.0
		**Change**	0.8 ± 19.0%	-10.6 ±28.9%	19.0 ± 29.8%	-5.4 ± 15.4%
	**Fibrate + Volanesorsen 300 mg (n = 1)**	**Pre**	11.6	241.0	47.0	178.0
		**Post**	3.0	91.0	63.5	75.0
		**Change**	-74.3%	-62.2%	35.1%	-57.9%
**E3/E3**	**Fibrate + Placebo (n = 1)**	**Pre**	10.3	204.0	37.0	178.0
		**Post**	15.3	314.0	30.0	179.0
		**Change**	48.4%	53.9%	-18.9%	0.6%
	**Fibrate + Volanesorsen 200 mg (n = 4)**	**Pre**	13.5 ± 1.0	262.1 ± 40.0	34.8 ± 3.8	188.0 ± 33.8
		**Post**	4.9 ± 1.9	126.5 ± 30.9	50.5 ± 10.1	145.4 ± 22.3
		**Change**	-63.8 ± 13.6%	-51.3 ± 11.3%	44.8 ± 21.0%	-21.8 ± 10.4%
	**Fibrate + Volanesorsen 300 mg (n = 3)**	**Pre**	15.7 ± 1.8	255.2 ± 17.1	39.7 ± 14.6	165.3 ± 47.0
		**Post**	5.5 ± 2.3	98.0 ± 30.4	61.2 ± 29.3	134.5 ± 19.0
		**Change**	-64.0 ± 16.5%	-61.0 ± 14.0%	51.1 ± 44.9%	-15.5 ± 18.0%
**E4/E4**	**Fibrate + Placebo (n = 1)**	**Pre**	25.5	495.0	30.0	316.0
		**Post**	21.4	408.0	31.0	281.0
		**Change**	-16.2%	-17.6%	3.3%	-11.1%
	**Fibrate + Volanesorsen 300 mg (n = 1)**	**Pre**	29.6	932.0	14.0	235.0
		**Post**	5.9	249.5	21.5	183.0
		**Change**	-80.1%	-73.2%	53.6%	-22.1%

Values represent mean ± SD.

Supplemental Table S5: Combined data of lipid analysis of a heterozygote apoE cohort administered with placebo or volanesorsen (Ionis 304801) as a monotherapy or add on to fibrates.

ApoE isoform	Treatment		Parameter<br>ApoC-III (mg/dL)	TGs (mg/dL)	HDL-C (mg/dL)	non-HDL-C (mg/dL)
**E2/E3**	**Placebo (n = 5)**	**Pre**	25.2 ± 10.7	760.2 ± 624.4	30.4 ± 10.9	241.2 ± 112.3
		**Post**	16.7 ± 4.4	489.0 ± 361.5	30.9 ± 10.5	182.6 ± 46.0
		**Change**	-23.4 ± 39.1%	-10.5 ± 78.5%	2.3 ± 10.7%	-15.2 ± 36.2%
	**Volanesorsen 100 mg (n = 2)**	**Pre**	12.0 ± 3.6	420.0 ± 174.0	37.0 ± 1.4	130.5 ± 55.9
		**Post**	9.4 ± 3.1	390.8 ± 161.6	35.8 ± 10.3	125.0 ± 20.5
		**Change**	-21.6 ± 2.0%	-7.0 ± 0.1%	-3.8 ± 24.0%	1.7 ± 27.8%
	**Volanesorsen 200 mg (n = 2)**	**Pre**	21.3 ± 12.0	594.0 ± 418.6	31.0 ± 4.2	199.5 ± 26.2
		**Post**	5.8 ± 0.1	166.5 ± 2.1	43.0 ± 0.7	200.5 ± 33.9
		**Change**	-67.5 ± 17.9%	-62.9 ± 25.8%	39.9 ± 16.9%	2.5 ± 30.5%
	**Volanesorsen 300 mg (n = 3)**	**Pre**	25.0 ± 5.8	491.3 ± 71.2	36.0 ± 2.0	204.7 ± 33.7
		**Post**	6.5 ± 2.9	164.8 ± 31.2	55.5 ± 3.5	123.3 ± 55.4
		**Change**	-74.8 ± 7.3%	-65.7 ± 9.9%	54.3 ± 9.0%	-40.9 ± 19.7%
**E2/E4**	**Placebo (n = 0)**	**Pre**	-	-	-	-
		**Post**	-	-	-	-
		**Change**	-	-	-	-
	**Volanesorsen 200 mg (n = 2)**	**Pre**	24.0 ± 2.8	715.8 ± 1.8	35.0 ± 4.2	178.0 ± 2.8
		**Post**	10.5 ± 10.3	424.5 ± 419.3	37.8 ± 12.4	176.5 ± 13.4
		**Change**	-53.3 ± 48.6%	-40.6 ± 58.7%	10.8 ± 48.8%	-0.8 ± 9.1%
**E3/E4**	**Placebo (n = 7)**	**Pre**	22.4 ± 2.8	489.6 ± 83.6	33.7 ± 5.4	176.7 ± 50.0
		**Post**	27.0 ± 13.7	671.3 ± 417.9	31.6 ± 5.1	188.6 ± 48.5
		**Change**	17.2 ± 44.1%	39.0 ± 88.4%	-5.7 ± 11.5%	15.1 ± 53.3%
	**Volanesorsen 100 mg (n = 6)**	**Pre**	23.2 ± 7.3	474.8 ± 158.8	31.7 ± 2.0	201.3 ± 30.3
		**Post**	14.0 ± 5.8	316.3 ± 158.0	38.1 ± 5.7	193.0 ± 52.4
		**Change**	-33.9 ± 37.3%	-18.6 ± 70.1%	20.0 ± 13.7%	-4.9 ± 20.4%
	**Volanesorsen 200 mg (n = 8)**	**Pre**	20.8 ± 6.4	533.4 ± 375.4	32.1 ± 5.4	194.6 ± 72.5
		**Post**	7.4 ± 3.5	188.6 ± 83.3	49.1 ± 18.8	163.1 ± 47.1
		**Change**	-61.0 ± 20.2%	-56.1 ± 24.3%	49.3 ± 32.4%	-11.7 ± 21.1%
	**Volanesorsen**	**Pre**	21.3 ± 7.4	520.7 ± 249.6	30.6 ± 10.8	147.6 ± 51.2

**300 mg (n = 7)**	**Post**	4.6 ± 2.0	155.8 ± 46.3	41.3 ± 9.3	168.2 ± 75.9
	**Change**	-75.4 ± 14.6%	-64.5 ± 15.5%	42.2 ± 28.0%	11.6 ± 29.3%

Values represent mean ± SD.

Supplemental Table S6: Lipid analysis of a heterozygote apoE cohort administered with placebo or volanesorsen (Ionis 304801) as a monotherapy.

ApoE isoform	Treatment		Parameter: ApoC-III (mg/dL)	TGs (mg/dL)	HDL-C (mg/dL)	non-HDL-C (mg/dL)
**E2/E3**	**Placebo (n = 3)**	**Pre**	29.6 ± 12.2	915.7 ± 786.0	28.7 ± 13.3	268.7 ± 147.7
		**Post**	17.8 ± 5.8	603.5 ± 458.4	28.2 ± 13.3	211.5 ± 12.1
		**Change**	-27.0 ± 53.7%	-1.0 ± 105.5%	-1.7 ± 7.1%	-4.6 ± 46.3%
	**Volanesorsen 100 mg (n = 2)**	**Pre**	12.0 ± 3.6	420.0 ± 174.0	37.0 ± 1.4	130.5 ± 55.9
		**Post**	9.4 ± 3.1	390.8 ± 161.6	35.8 ± 10.3	125.0 ± 20.5
		**Change**	-21.6 ± 2.0%	-7.0 ± 0.1%	-3.8 ± 24.0%	1.7 ± 27.8%
	**Volanesorsen 200 mg (n = 2)**	**Pre**	21.3 ± 12.0	594.0 ± 418.6	31.0 ± 4.2	199.5 ± 26.2
		**Post**	5.8 ± 0.1	166.5 ± 2.1	43.0 ± 0.7	200.5 ± 33.9
		**Change**	-67.2 ± 17.9%	-62.9 ± 25.8%	39.9 ± 16.9%	2.5 ± 30.5%
	**Volanesorsen 300 mg (n = 1)**	**Pre**	30.3	573.0	38.0	180.0
		**Post**	8.6	151.5	55.5	118.5
		**Change**	-71.6%	-73.6%	46.1%	-34.2%
**E2/E4**	**Placebo (n = 0)**	**Pre**	-	-	-	-
		**Post**	-	-	-	-
		**Change**	-	-	-	-
	**Volanesorsen 200 mg (n = 2)**	**Pre**	24.0 ± 2.8	715.8 ± 1.8	35.0 ± 4.2	178.0 ± 2.8
		**Post**	10.5 ± 10.3	424.5 ± 419.3	37.8 ± 12.4	176.5 ± 13.4
		**Change**	-53.3 ± 48.6%	-40.6 ± 58.7%	10.8 ± 48.8%	-0.8 ± 9.1%
**E3/E4**	**Placebo (n = 6)**	**Pre**	22.2 ± 3.0	495.4 ± 90.0	33.3 ± 5.8	184.3 ± 50.1
		**Post**	28.3 ± 14.5	719.8 ± 435.7	31.5 ± 5.6	201.6 ± 37.4
		**Change**	22.7 ± 45.5%	48.2 ± 93.0%	-4.8 ± 12.3%	20.2 ± 56.5%
	**Volanesorsen 100 mg (n = 6)**	**Pre**	23.2 ± 7.3	474.8 ± 158.8	31.7 ± 2.0	201.3 ± 30.3
		**Post**	14.0 ± 5.8	316.3 ± 158.0	38.1 ± 5.7	193.0 ± 52.4
		**Change**	-33.9 ± 37.3%	-18.6 ± 70.1%	20.0 ± 13.7%	-4.9 ± 20.5%
	**Volanesorsen 200 mg (n = 5)**	**Pre**	23.1 ± 6.4	667.0 ± 423.8	31.6 ± 3.0	227.8 ± 69.3
		**Post**	7.3 ± 3.8	205.5 ± 71.3	45.6 ± 10.2	186.9 ± 42.0
		**Change**	-64.4 ± 24.7%	-59.3 ± 28.6%	43.9 ± 24.6%	-14.9 ± 16.3%
	**Volanesorsen 300 mg**	**Pre**	22.4 ± 8.5	563.2 ± 259.6	32.0 ± 11.1	134.2 ± 44.8
		**Post**	4.0 ± 1.8	161.3 ± 43.7	40.6 ± 7.0	154.7 ± 62.0

**(n = 5)**	**Change**	-79.9 ± 11.4%	-65.0 ± 18.6%	34.3 ± 29.7%	13.9 ± 32.2%

Values represent mean ± SD.

Supplemental Table S7: Lipid analysis of a heterozygote apoE cohort administered with placebo or volanesorsen (Ionis 304801) as add on to fibrates.

ApoE isoform	Treatment		Parameter<br>ApoC-III (mg/dL)	TGs (mg/dL)	HDL-C (mg/dL)	non-HDL-C (mg/dL)
E2/E3	Fibrate + Placebo (n = 2)	Pre	18.6 ± 3.4	527.0 ± 377.6	33.0 ± 9.9	200.0 ± 33.9
		Post	15.0 ± 0.1	317.3 ± 64.0	35.0 ± 5.7	139.3 ± 43.5
		Change	-17.8 ± 15.7%	-24.9 ± 41.7%	8.4 ± 15.4%	-31.2 ± 10.1%
	Fibrate + Volanesorsen 300 mg (n = 2)	Pre	22.3 ± 5.0	450.5 ± 12.0	35.0 ± 1.4	217.0 ± 36.8
		Post	5.5 ± 3.3	171.5 ± 41.0	55.5 ± 5.0	125.8 ± 78.1
		Change	-76.5 ± 9.5%	-61.8 ± 10.1%	58.4 ± 7.7%	-44.3 ± 26.6%
E2/E4	Fibrate + Placebo (n = 0)	Pre	-	-	-	-
		Post	-	-	-	-
		Change	-	-	-	-
	Fibrate + Volanesorsen (n = 0)	Pre	-	-	-	-
		Post	-	-	-	-
		Change	-	-	-	-
E3/E4	Fibrate + Placebo (n = 1)	Pre	23.4	455.0	36.0	131.0
		Post	19.6	380.5	32.0	110.5
		Change	-16.2%	-16.4%	-11.1%	-15.6%
	Fibrate + Volanesorsen 200 mg (n = 3)	Pre	16.9 ± 5.2	310.7 ± 122.3	33.0 ± 9.0	139.3 ± 37.8
		Post	7.7 ± 3.7	160.5 ± 110.5	54.8 ± 30.8	123.3 ± 22.5
		Change	-55.3 ± 11.5%	-50.7 ± 18.8%	58.3 ± 47.6%	-6.4 ± 31.0%
	Fibrate + Volanesorsen 300 mg (n = 2)	Pre	18.5 ± 4.5	414.5 ± 269.4	27.0 ± 12.7	181.0 ± 67.9
		Post	6.2 ± 2.1	142.0 ± 68.6	43.0 ± 17.7	202.0 ± 126.6
		Change	-64.2 ± 20.0%	-63.4 ± 7.3%	61.8 ± 10.8%	5.9 ± 30.2%

Values represent mean ± SD.

Supplemental Figures:

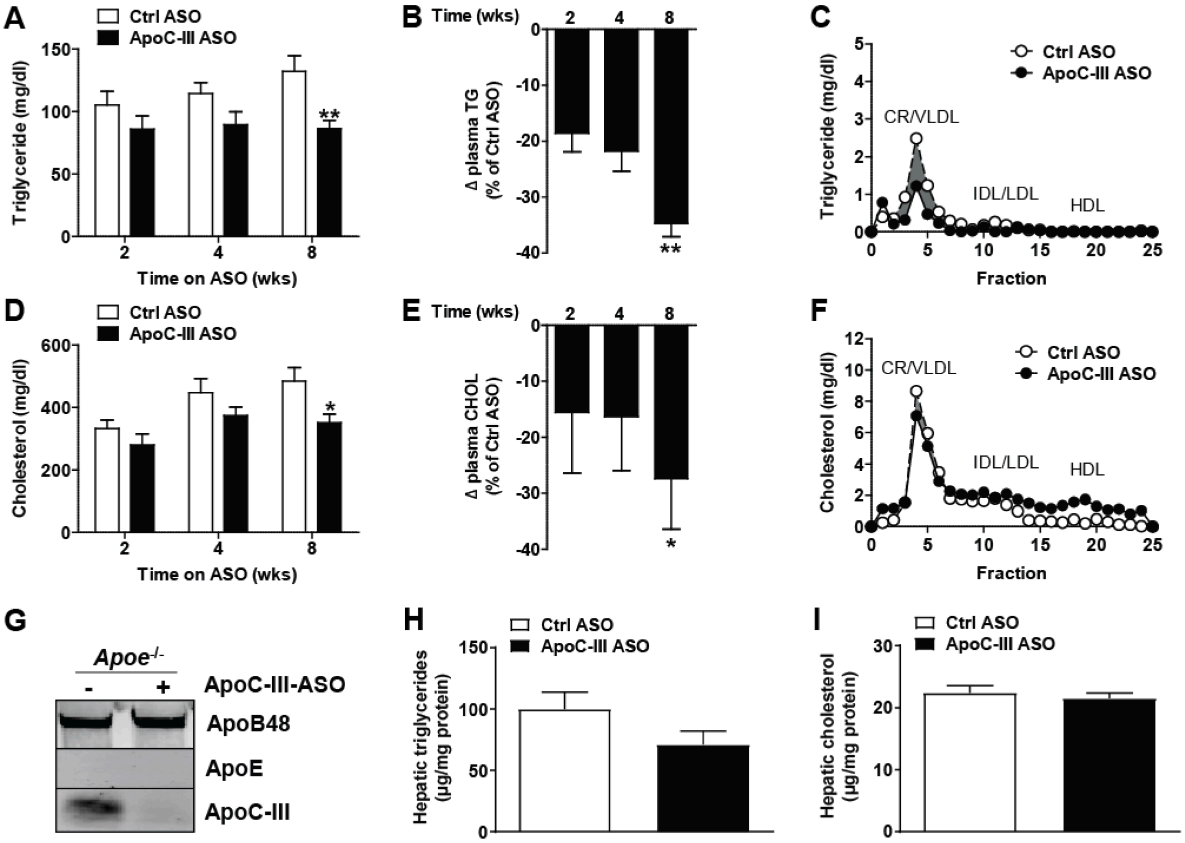

Supplemental Figure S1. Targeting *apoC-III* with ASOs in chow-fed *Apoe*$^{-/-}$ mice. (**A**) *Apoe*$^{-/-}$ mice were administered once weekly with apoC-III ASO or control ASO (50 mg/kg bodyweight) for a period of 8 weeks. Fasting plasma TG levels were measured at the indicated time points. (**B**) ApoC-III ASO mediated relative change of plasma TG levels compared with control ASO. (**C**) Pooled plasma samples after 4 weeks of ASO treatment were analyzed by size-exclusion FPLC and TGs in each fraction were measured. (**D**) Fasting plasma cholesterol levels after apoC-III ASO treatment and (**E**) relative change in cholesterol compared to control ASO. (**F**) FPCL analysis of pooled plasma samples 4 weeks of ASO treatment followed by cholesterol determination in each fraction. (**G**) VLDLs were isolated by ultracentrifugation and pooled VLDL samples (5 µg protein/lane) were analyzed by Western blotting with antibodies against apoB, apoC-III and apoE. Hepatic TG (**H**) and cholesterol (**I**) levels were measured (n = 8-10/group, values represent mean ± SEM, *p<0.05, **p<0.01).

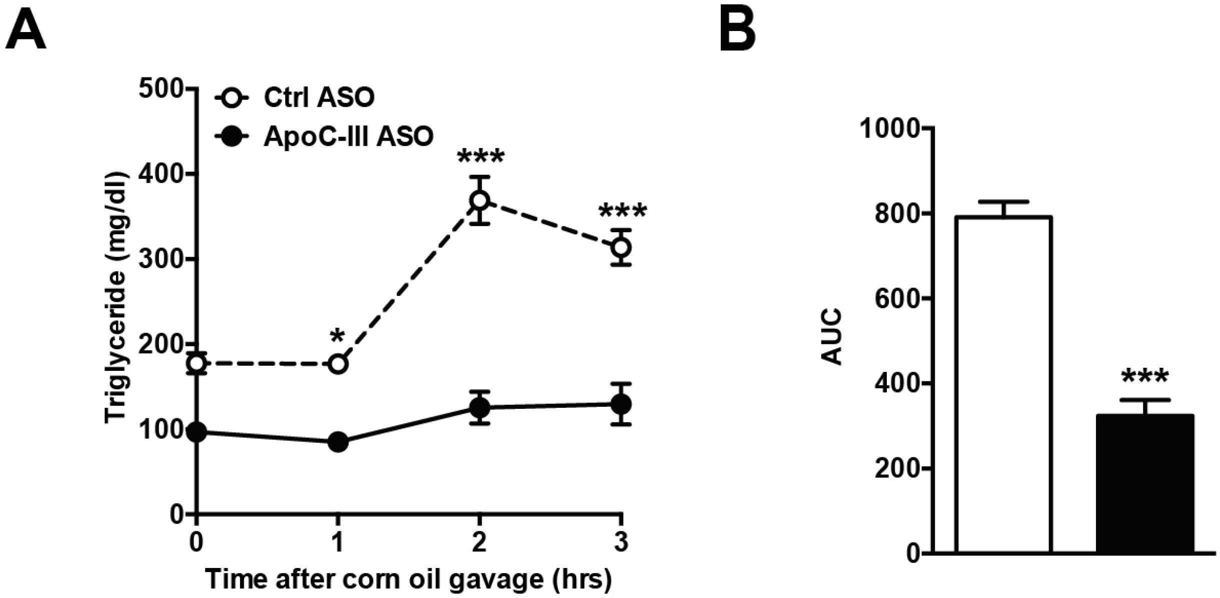

Supplemental Figure S2. Fat Tolerance Test in Western Diet Fed *Apoe$^{-/-}$Ndst1$^{f/f}$Alb-Cre^{+}* mice. (A-B) ApoC-III ASO or control ASO (50 mg/kg bodyweight) were administered to *Apoe$^{-/-}$Ndst1$^{f/f}$Alb-Cre^{+}* mice fed a Western-type diet for 8 weeks and *Apoe$^{-/-}$Ndst1$^{f/f}$Alb-Cre^{+}* mice. Fasted mice (5h) were given a 250 µL bolus of corn oil by oral gavage to measure fat tolerance. Postprandial TG levels were measured 0, 1, 2, and 3 hours after gavage (n = 4/group).

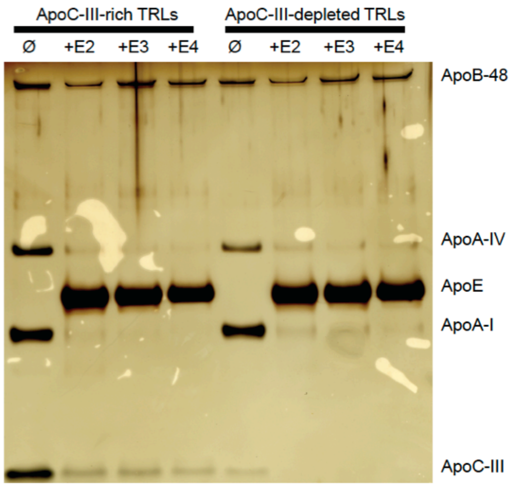

Supplemental Figure S3: ApoE isoform reconstitution of murine apoE-deficient TRLs. Silver stained gel of [^{3}H]TRLs reconstituted with either apoE2, E3 or E4 (apoE to [^{3}H]TRL ratio 1:2).

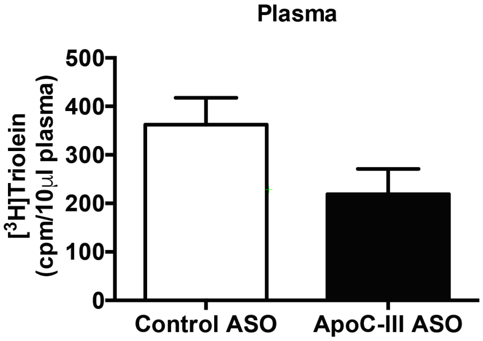

Supplemental Figure S4. ApoC-III lowering decreased [^{3}H]triolein labeled liposyn particles in *Apoe$^{-/-}$Ndst1$^{f/f}$Alb-Cre$^+$* mice. *Apoe$^{-/-}$Ndst1$^{f/f}$Alb-Cre$^+$* mice were fasted for 5 h. After a 5 min intravenous injection of [^{3}H]triolein labeled liposyn particles (100 µl) blood was collected. [^{3}H]triolein plasma concentrations were determined by liquid scintillation counting (n = 4-6).
